# Predictors of cardio-kidney complications and treatment failure in patients with chronic kidney disease and type 2 diabetes treated with SGLT2 inhibitors

**DOI:** 10.1186/s12916-021-02191-2

**Published:** 2022-01-10

**Authors:** Csaba Kovesdy, Niklas Schmedt, Kerstin Folkerts, Kevin Bowrin, Hanaya Raad, Michael Batech, Linda Fried

**Affiliations:** 1 Division of Nephrology, Department of Medicine, University of Tennessee, Memphis, TN USA; 2 Bayer AG, Berlin, Germany; 3 Bayer AG, Wuppertal, Germany; 4 Bayer Plc, Reading, UK; 5 Science, Aetion, Inc., Paris, France; 6 Science, Aetion, Inc., Frankfurt am Main, Germany; 7 7E121 VA Pittsburgh Healthcare System, University of Pittsburgh, University Drive Center, Pittsburgh, PA 15240 USA

**Keywords:** Type 2 diabetes, Chronic kidney disease, Treatment failure, Outcomes, Predictors

## Abstract

**Background:**

Clinical practice guidelines recommend sodium-glucose co-transporter 2 inhibitors (SGLT2is) to mitigate adverse kidney and cardiovascular outcomes in patients with type 2 diabetes (T2D), including patients with comorbid chronic kidney disease (CKD), also referred to as diabetic kidney disease (DKD), who are at even higher risk. In this study, we sought to identify predictors of cardio-kidney events, cardio-kidney complications, and treatment failure (i.e., addition/initiation of a new T2D class, insulin, or discontinuation of SGLT2is) after new initiation of SGLT2is in patients with CKD and T2D (DKD).

**Methods:**

In this retrospective cohort study, we identified adult patients with DKD who initiated SGLT2is between April 1, 2012, and June 30, 2019, in Optum claims data. Outcome rates per 1000 person-years (PY) are reported with 95% confidence intervals (CIs). Cox proportional hazards regression identified patient characteristics associated with each outcome.

**Results:**

The study population consisted of 6389 initiators of SGLT2is. The rate of CV hospitalization was 26.0 (95% CI 21.6, 30.4) per 1000 PY. Baseline characteristics associated with higher risk of CV hospitalization included age, atrial fibrillation, peripheral vascular disease (PVD), and cancer. The rate of kidney hospitalization was 12.0 (95% CI 9.0, 15.0) per 1000 PY. The risk increased significantly with baseline evidence of heart failure, hyperkalemia, respiratory failure, depression, and use of loop diuretics. In total, 55.0% of all SGLT2i initiators discontinued treatment during the follow-up period. The rate of treatment failure was 510.5 (95% CI 492.9, 528.1) per 1000 PY. Analysis of key time-dependent SGLT2i-associated adverse events showed that experiencing diabetic ketoacidosis and volume depletion were associated with risk of treatment failure.

**Conclusions:**

Our study demonstrated high rates of residual cardio-kidney outcomes and treatment failure in patients with DKD treated with SGLT2is. Patients with high baseline CV risk and the presence of certain conditions, such as atrial fibrillation, PVD, and heart failure, were at higher risk for cardio-kidney events. Further research is needed to assess the potential relationship between adverse events and SGLT2i treatment failure.

**Supplementary Information:**

The online version contains supplementary material available at 10.1186/s12916-021-02191-2.

## Background

Chronic kidney disease (CKD) is a serious complication of type 2 diabetes (T2D) that afflicts between 25 and 40% of all diagnosed patients [1]. CKD progression differs by factors such as age [2], gender, and genetic [3] characteristics and entails compromised kidney function or kidney injury attributed to persistent uncontrolled hyperglycemia, the presence and/or release of pro-inflammatory proteins and pro-fibrotic proteins, and hypertension or high intra-glomerular pressure associated with elevated rates of cardiovascular disease (CVD) compared to T2D alone [4]. This elevated cardiovascular (CV) risk is partially responsible for the heightened mortality risk among patients with CKD, which is markedly higher compared to the general population or patients with T2D alone [5].

Prolonged, unmanaged CKD in T2D, also referred to as diabetic kidney disease (DKD), often leads to end-stage kidney disease (ESKD), which requires dialysis and/or need for kidney transplantation [6, 7]. These procedures, in addition to hospitalizations common with ESKD patients, represent a severe strain on health care systems, reaching costs of more than $35 billion in the United States (US) in 2017 alone, accounting for 7.2% of overall Medicare paid claims and a share which has remained nearly unchanged for over a decade [8].

There is an increasing need for new therapeutic agents that may slow or halt the progression of DKD and reduce the high morbidity and mortality in this population. Sodium-glucose co-transporter 2 inhibitors (SGLT2is), the most recently approved class of glucose-lowering agents for T2D in the US in March 2013, have later shown beneficial effects on kidney and cardiovascular outcomes and are recommended in clinical practice guidelines [9, 10]. Notwithstanding the substantial clinical benefit of SGLT2is shown in clinical trials, real-life evidence on the remaining unmet need and treatment patterns among patients with DKD prescribed SGLT2is are scarce [11, 12]. Despite the known protective cardio-kidney effects of SGLT2is, many patients still experience cardiovascular and kidney complications or treatment failure [13]. Prior research has shown that one quarter of patients with T2D prescribed SGLT2is discontinue their treatment within the first year after newly initiating treatment [14]. The role of safety concerns in SGLT2i treatment discontinuation is unclear; side effects of SGLT2is include genital infections, increased urination, weakness, and lethargy, which are mostly mild to moderate and rarely lead to discontinuation [15]. Determining the factors associated with cardio-kidney complications and treatment failure after the initiation of SGLT2is may help to better understand the overall unmet need of SGLT2i-treated patients despite the availability of this risk-reducing medication. To better understand these factors, we sought to identify predictors of cardio-kidney events and predictors of treatment failure among initiators of SGLT2i with DKD using US-based real-world data.

## Methods

### Study design and setting

This retrospective observational cohort study was conducted using Optum Clinformatics™ Data Mart (CDM) between April 1, 2012, to June 30, 2019, to identify predictors of cardio-kidney events and predictors of treatment failure among initiators of SGLT2i with DKD. The data source is an administrative health claims database with longitudinal data of patients enrolled in a commercial and Medicare Advantage health plan data in the US. The data contain demographic, medical encounters from inpatient and outpatient settings, pharmacy dispensing, and laboratory results for a subset of patients. The data contain approximately 63 million unique members and are considered representative of the commercially insured US population.

### Study population

**Fig. 1:**
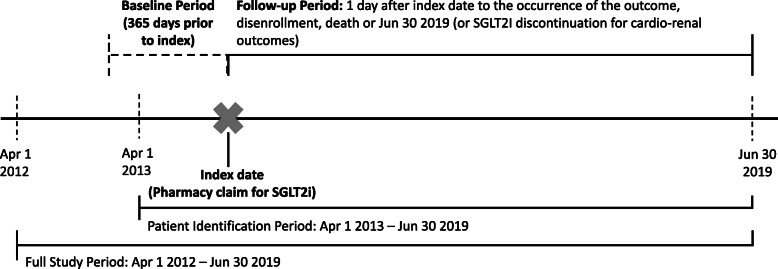
Study timeline diagram. Abbreviation: SGLT2i = sodium-glucose co-transporter-2 inhibitor

The study population consisted of patients aged 18 years or older with DKD who initiated SGLT2i therapy following a 365-day SGLT2i-naïve period and 365-day continuous enrollment period. The first pharmacy claim for SGLT2i between 1 April 2013 (following approval of SGLT2is in the US) and 30 June 2019 was considered the index date**.** Follow-up period began 1 day after the index date and ended on the earliest occurrence of the outcome, disenrollment, death, and the end of data on 30 June 2019 (or SGLT2i discontinuation for cardio-kidney outcomes only) (Fig.1). T2D diagnosis was defined according to at least one inpatient ICD-9 or ICD-10 diagnosis code in any position, at least two outpatient ICD-9 and ICD-10 diagnosis codes for T2D in any position 30 to 365 days apart, or at least one prescription claim for a second-line therapy agent (i.e., sulfonylureas, thiazolidinedione (TZD), dipeptidyl peptidase-4 inhibitor (DPP4i), glucagon-like peptide-1 receptor agonist (GLP1ra), insulin, meglitinide, or alpha glucosidase inhibitors) for T2D in the baseline period [16]. CKD diagnosis was defined according to two laboratory values for estimated glomerular rate (eGFR) and/or two laboratory values for urine albumin-to-creatinine ratio (UACR) 90 to 365 days apart within ranges indicating CKD in the baseline period. Patients with baseline claims for conditions other than T2D that may cause kidney disease, such as glomerulonephritis, membranous nephropathy, or malignant kidney tumor were excluded [17]. Full definitions of the patient cohorts, variables, outcomes, and subgroup selection criteria are listed in Additional File 1: Table S1.

### Variables and outcomes

Patient demographic characteristics were assessed on index date. Comorbidities, laboratory values (e.g., eGFR, UACR, and hemoglobin A1c [HbA1c] values closest to the index date), and medication use (cardiovascular and antiglycemic agents) were assessed during the 365-day baseline period.

Cardio-kidney events and their predictors were evaluated separately in the study population during the follow-up period. These included CV hospitalization (defined by unstable or stable angina, arrhythmia, coronary intervention or revascularization, transient ischemic attack, heart failure, and peripheral arterial disease), kidney hospitalization defined by CKD diagnosis, and AKI hospitalization. Treatment failure was also evaluated during follow-up, defined as the occurrence of discontinuation of SGLT2i or switch from SGLT2i to another T2D medication class; or addition of another T2D medication class not used in baseline; or initiation of insulin (see Additional File 1: Table S1 for a complete listing of all treatments considered). We applied a conservative grace period of 90 days between two consecutive refills before counting SGLT2i treatment as discontinued [18].

### Statistical analysis

The number and percentage of patients meeting each characteristic were reported. Means (with standard deviations) and medians (with interquartile ranges reported as 25th and 75th percentiles; IQR) were reported for continuous characteristics. Baseline medication use of patients was described according to proportion of patients with at least one dispensation of a drug of interest. Event rates of each outcome per 1000 person-years (PY) were reported, and 95% confidence intervals (CIs) were estimated using Poisson regression with a Normal approximation. For the rate calculations, the numerator consisted of the total number of first occurrences in the population and the denominator included the total person-time (in years) from 1 day after the index date to the date of the outcome or the end of follow-up.

Cox proportional hazard regression was used to identify baseline patient characteristics associated with an increased risk for the outcomes of interest. All results were reported as hazard ratios (HRs) with 95% CIs. Two-sided *p* values were reported with a pre-specified alpha (*ɑ*) level of 0.05. Backward selection methods were used to identify a subset of covariates associated with each outcome [19]. Multivariate models including all input variables were initially fitted and then reduced in a stepwise manner to identify the best fit according to the Akaike information criterion (AIC). Prior to the initial model fitting, variables with high collinearity and correlation were removed from the total list of input variables; tests of relative importance were applied when necessary.

### Subgroup and sensitivity analyses

All baseline analyses were stratified by insurance type due to the potential differences in T2D management based on commercial versus Medicare insurance plans. Several supplemental analyses were also performed, including the analysis of cardio-kidney outcomes by insurance type subgroups (i.e., commercial or Medicare Advantage; Additional File 2: Figures S1 – S4). Additionally, all baseline characteristics and the CV outcome were analyzed in mutually exclusive cardiovascular risk subgroups based on the level of CVD risk in the baseline period as follows: (1) patients with no inpatient or outpatient CVD diagnosis in baseline (low risk), (2) patients with an outpatient but no inpatient CVD diagnosis in baseline (moderate risk), and (3) patients with inpatient CVD diagnosis in baseline (high risk).

An additional sensitivity analysis explored the effects of several time-dependent SGLT2i-related adverse events (AE) after treatment initiation on treatment failure. These included acidosis, hospitalization for acute kidney injury (AKI), dehydration, diabetic ketoacidosis, genital tract infection, hypotension, lower extremity amputation, urinary tract infections (UTI), and volume depletion. Each of these events was evaluated in 30-day risk-interval windows after the index date until the time of censoring. Baseline factors from the final multivariate treatment failure model along with the time-dependent events of interest were explored univariately and final multivariate models were explored.

All analyses were conducted using the Aetion Evidence Platform® (2020), software for real-world data analysis, which is validated for a range of studies [20].

## Results

**Fig. 2:**
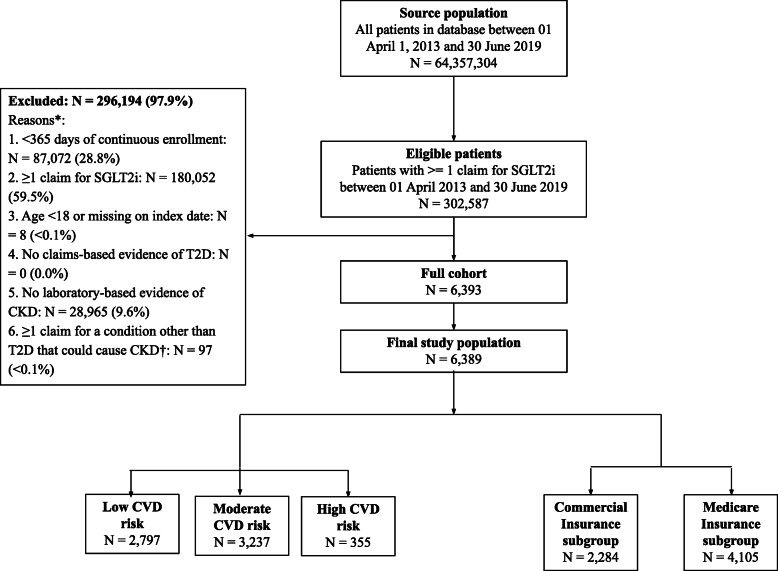
Flowchart of patient selection for study population. * All exclusion criteria were assessed during the 365-day baseline period unless otherwise noted. † Conditions other than T2D that could cause DKD included the following: glomerulonephritis, focal glomerulosclerosis/focal sclerosing glomerulonephritis, membranous nephropathy, membranoproliferative glomerulonephritis type 1/diffuse membranoproliferative glomerulosclerosis, immunoglobin A nephropathy/Berger’s disease, rapidly progressive, systemic lupus erythematosus nephritis, glomerulonephritis, other proliferative glomerulonephritis, Wegener’s granulomatosis, other vasculitis with kidney involvement, interstitial nephritis/pyelonephritis from analgesic abuse, gouty nephropathy, acquired obstructive uropathy, chronic pyelonephritis/reflux nephropathy, chronic interstitial nephritis, acute interstitial nephritis, urolithiasis, renal artery stenosis, renal artery occlusion, adult polycystic kidney disease, malignant renal tumor, multiple myeloma, acquired immunodeficiency syndrome, nephropathy and tubular necrosis, renal agenesis, dysgenesis, hypoplasia, and sickle cell disease. Abbreviations: CDM = Clinformatics Data Mart, DKD = diabetic kidney disease, SGLT2i = sodium-glucose co-transporter-2 inhibitor, T2D = type 2 diabetes

The study population consisted of 6389 patients with DKD at baseline who newly initiated SGLT2is patients. Figure 2 presents the study population inclusion and exclusion summary. Subgroups included 2284 patients with commercial insurance and 4105 patients with Medicare insurance, as well as 2797 patients with low CV risk, 3237 patients with moderate CV risk, and 355 patients with high CV risk.

### Patient characteristics

Table 1 summarizes the patient baseline characteristics. SGLT2i initiators were on average 65.5 (SD 10.6) years of age with an even distribution of use in males and females. CV risk subgroups had similar average age while Medicare enrollees were on average 70.6 (SD7.8) and commercially insured patients were 56.4 (8.7) years of age. Approximately half of all the patients were White; 59.1% of patients resided in the South.

**Table 1:** Demographics of SGLT2i initiators with DKD, in the total study population, and stratified by insurance type and CV risk

Demographics	SGLT2i initiators with DKD (***N*** = 6389)	Commercial insurance (***N*** = 2284)	Medicare insurance (***N*** = 4105)	Low CV risk (***N*** = 2797)	Moderate CV risk (***N*** = 3237)	High CV risk (***N*** = 355)
Age						
Mean (SD)	65.5 (10.6)	56.4 (8.7)	70.6 (7.8)	64.4 (10.6)	66.4 (10.5)	66.7 (10.3)
Median [IQR]	67 [59, 73]	58 [51, 62]	71 [67, 75]	66 [57, 72]	68 [60, 73]	68 [61, 74]
Sex						
Female, *n* (%)	3006 (47.0%)	880 (38.5%)	2126 (51.8%)	1341 (47.9%)	1510 (46.6%)	155 (43.7%)
Male, *n* (%)	3382 (52.9%)	1403 (61.4%)	1979 (48.2%)	1455 (52.0%)	1727 (53.4%)	200 (56.3%)
Unknown, *n* (%)	1 (0.0%)	1 (0.0%)	0 (0.0%)	1 (0.0%)	0 (0.0%)	0 (0.0%)
Race						
White, *n* (%)	3062 (47.9%)	1297 (56.8%)	1765 (43.0%)	1486 (53.1%)	1416 (43.7%)	160 (45.1%)
Asian, *n* (%)	419 (6.6%)	145 (6.3%)	274 (6.7%)	190 (6.8%)	214 (6.6%)	15 (4.2%)
Black, *n* (%)	773 (12.1%)	267 (11.7%)	506 (12.3%)	337 (12.0%)	381 (11.8%)	55 (15.5%)
Hispanic, *n* (%)	1484 (23.2%)	469 (20.5%)	1015 (24.7%)	515 (18.4%)	879 (27.2%)	90 (25.4%)
Missing, *n* (%)	651 (10.2%)	106 (4.6%)	545 (13.3%)	269 (9.6%)	347 (10.7%)	35 (9.9%)
Region						
Northeast, *n* (%)	564 (8.8%)	155 (6.8%)	409 (10.0%)	235 (8.4%)	285 (8.8%)	44 (12.4%)
Midwest, *n* (%)	433 (6.8%)	235 (10.3%)	198 (4.8%)	236 (8.4%)	166 (5.1%)	31 (8.7%)
South, *n* (%)	3779 (59.1%)	1379 (60.4%)	2400 (58.5%)	1616 (57.8%)	1937 (59.8%)	226 (63.7%)
West, *n* (%)	1584 (24.8%)	511 (22.4%)	1073 (26.1%)	700 (25.0%)	832 (25.7%)	52 (14.6%)
Missing, *n* (%)	29 (0.5%)	4 (0.2%)	25 (0.6%)	10 (0.4%)	17 (0.5%)	2 (0.6%)
Provider specialty						
Endocrinologist, *n* (%)	469 (7.3%)	206 (9.0%)	263 (6.4%)	145 (5.2%)	292 (9.0%)	32 (9.0%)
Nephrologist, *n* (%)	35 (0.5%)	12 (0.5%)	23 (0.6%)	14 (0.5%)	17 (0.5%)	4 (1.1%)
Cardiologist, *n* (%)	91 (1.4%)	24 (1.1%)	67 (1.6%)	36 (1.3%)	43 (1.3%)	12 (3.4%)
GP/Internist, *n* (%)	1556 (24.4%)	424 (18.6%)	1132 (27.6%)	645 (23.1%)	825 (25.5%)	86 (24.2%)
Urologist, *n* (%)	12 (0.2%)	2 (0.1%)	10 (0.2%)	5 (0.2%)	6 (0.2%)	1 (0.3%)
IP facility provider, *n* (%)	72 (1.1%)	27 (1.2%)	45 (1.1%)	22 (0.8%)	39 (1.2%)	11 (3.1%)
OP facility provider, *n* (%)	61 (1.0%)	12 (0.5%)	49 (1.2%)	22 (0.8%)	36 (1.1%)	3 (0.8%)
Missing, *n* (%)	2067 (32.4%)	635 (27.8%)	1432 (34.9%)	820 (29.3%)	1122 (34.7%)	125 (35.2%)
Insurance type						
Commercial, *n* (%)	2284 (35.7%)	2284 (100.0%)	0 (0.0%)	1234 (44.1%)	957 (29.6%)	93 (26.2%)
Medicare, *n* (%)	4105 (64.3%)	0 (0.0%)	4105 (100.0%)	1563 (55.9%)	2280 (70.4%)	262 (73.8%)

Abbreviations: *CDM* Clinformatics Data Mart, *DKD* diabetic kidney disease, *CV* cardiovascular, *GP* general practitioner, *IP* inpatient, *IQR* interquartile range, *OP* outpatient, *SD* standard deviation, *SGLT2i* sodium-glucose co-transporter-2 inhibitor, *T2D* type 2 diabetes

The most prevalent comorbidities (Table 2) in the study population were hypertension (92.3%), hyperlipidemia (87.9%), pain disorders (70.2%), microvascular complications (54.3%), and obesity (43.2%). The average HbA1c level in the study population was 9.2% (SD 3.9) at baseline and ranged from 8.9% (SD 3.6) to 9.4% (SD 4.3) among subgroups. Approximately a third of patients (31.7%) had mild-to-moderately decreased eGFR (stage G3a), the highest percentage being in the high CV risk subgroup (36.1%) and in Medicare patients (35.0%). 43.4% had moderately increased albuminuria (UACR stage A2). Less than 2% of patients had decreased eGFR or kidney failure at baseline (eGFR stage of G4 or G5, respectively). The mean annual rate of eGFR decline during follow-up was 4.4 (SD 6.8) ml/min/1.73 m^2^ per year for patients with treatment failure and 8.7 (SD 13.6) ml/min/1.73 m^2^ per year for patients with cardio-kidney events increasing to up to 12.4 (SD 13.1) among patients with high CV risk.

**Table 2:** Baseline comorbidities, lifestyle factors and lab tests among SGLT2i initiators with DKD, in the total study population and stratified by insurance type and CV risk

	SGLT2i initiators with DKD (***N*** = 6389)	Commercial insurance (***N*** = 2284)	Medicare insurance (***N*** = 4105)	Low CV risk (***N*** = 2797)	Moderate CV risk (***N*** = 3237)	High CV risk (***N*** = 355)
**Comorbidities**						
DSCI score						
Mean (SD)	2.5 (2.0)	1.6 (1.7)	2.9 (2.0)	1.7 (1.5)	3.0 (2.1)	4.5 (2.0)
Median [IQR]	2 [1, 4]	1 [0, 2]	3 [1, 4]	1 [0, 3]	3 [1, 4]	4 [3, 6]
Charlson-Quan score						
Mean (SD)	1.6 (1.8)	1.5 (1.7)	1.6 (1.9)	1.7 (1.8)	1.4 (1.8)	2.5 (2.1)
Median [IQR]	1 [0, 2]	1 [0, 2]	1 [0, 2]	1 [0, 3]	1 [0, 2]	2 [1, 3]
Acidosis, *n* (%)	89 (1.4%)	21 (0.9%)	68 (1.7%)	25 (0.9%)	34 (1.1%)	30 (8.5%)
ACS, *n* (%)	352 (5.5%)	73 (3.2%)	279 (6.8%)	37 (1.3%)	196 (6.1%)	119 (33.5%)
Angina pectoris, *n* (%)	1671 (26.2%)	397 (17.4%)	1274 (31.0%)	462 (16.5%)	955 (29.5%)	254 (71.5%)
Atherosclerotic gangrene, *n* (%)	9 (0.1%)	2 (0.1%)	7 (0.2%)	0 (0.0%)	1 (0.0%)	8 (2.3%)
Atrial fibrillation, *n* (%)	87 (1.4%)	19 (0.8%)	68 (1.7%)	24 (0.9%)	22 (0.7%)	41 (11.5%)
CABG or PTCA, n (%)	146 (2.3%)	45 (2.0%)	101 (2.5%)	10 (0.4%)	43 (1.3%)	93 (26.2%)
Cancer excluding NMSC, *n* (%)	488 (7.6%)	128 (5.6%)	360 (8.8%)	213 (7.6%)	243 (7.5%)	32 (9.0%)
COPD, *n* (%)	1271 (19.9%)	306 (13.4%)	965 (23.5%)	423 (15.1%)	709 (21.9%)	139 (39.2%)
Coronary artery disease, *n* (%)	1437 (22.5%)	308 (13.5%)	1129 (27.5%)	296 (10.6%)	895 (27.6%)	246 (69.3%)
Depression, *n* (%)	955 (14.9%)	237 (10.4%)	718 (17.5%)	302 (10.8%)	565 (17.5%)	88 (24.8%)
Diabetic foot infection, *n* (%)	304 (4.8%)	98 (4.3%)	206 (5.0%)	81 (2.9%)	160 (4.9%)	63 (17.7%)
Diabetic retinopathy, *n* (%)	1107 (17.3%)	317 (13.9%)	790 (19.2%)	432 (15.4%)	601 (18.6%)	74 (20.8%)
Edema, *n* (%)	833 (13.0%)	218 (9.5%)	615 (15.0%)	277 (9.9%)	446 (13.8%)	110 (31.0%)
Fatigue/sleep disorder, *n* (%)	1571 (24.6%)	479 (21.0%)	1092 (26.6%)	572 (20.5%)	823 (25.4%)	176 (49.6%)
GERD, *n* (%)	1308 (20.5%)	345 (15.1%)	963 (23.5%)	465 (16.6%)	705 (21.8%)	138 (38.9%)
Heart failure, *n* (%)	620 (9.7%)	106 (4.6%)	514 (12.5%)	142 (5.1%)	345 (10.7%)	133 (37.5%)
Hyperkalemia, *n* (%)	348 (5.4%)	98 (4.3%)	250 (6.1%)	116 (4.1%)	172 (5.3%)	60 (16.9%)
Hyperlipidemia, *n* (%)	5615 (87.9%)	2020 (88.4%)	3595 (87.6%)	2398 (85.7%)	2881 (89.0%)	336 (94.6%)
Hypertension, *n* (%)	5897 (92.3%)	2060 (90.2%)	3837 (93.5%)	2506 (89.6%)	3040 (93.9%)	351 (98.9%)
Hypoglycemia, *n* (%)	129 (2.0%)	31 (1.4%)	98 (2.4%)	31 (1.1%)	69 (2.1%)	29 (8.2%)
Hypokalemia, *n* (%)	193 (3.0%)	65 (2.8%)	128 (3.1%)	46 (1.6%)	108 (3.3%)	39 (11.0%)
Hyponatremia, *n* (%)	146 (2.3%)	25 (1.1%)	121 (2.9%)	32 (1.1%)	63 (1.9%)	51 (14.4%)
Hypotension, *n* (%)	180 (2.8%)	49 (2.1%)	131 (3.2%)	45 (1.6%)	78 (2.4%)	57 (16.1%)
Infectious disease, *n* (%)	1673 (26.2%)	465 (20.4%)	1208 (29.4%)	546 (19.5%)	969 (29.9%)	158 (44.5%)
Intestinal enteropathy, *n* (%)	744 (11.6%)	197 (8.6%)	547 (13.3%)	242 (8.7%)	402 (12.4%)	100 (28.2%)
Liver disease, *n* (%)	508 (8.0%)	185 (8.1%)	323 (7.9%)	182 (6.5%)	283 (8.7%)	43 (12.1%)
MC disease, *n* (%)	3467 (54.3%)	955 (41.8%)	2512 (61.2%)	1306 (46.7%)	1922 (59.4%)	239 (67.3%)
Obesity, *n* (%)	2759 (43.2%)	1006 (44.0%)	1753 (42.7%)	980 (35.0%)	1567 (48.4%)	212 (59.7%)
Osteoarthritis, *n* (%)	1483 (23.2%)	359 (15.7%)	1124 (27.4%)	592 (21.2%)	758 (23.4%)	133 (37.5%)
Pain disorders, *n* (%)	4487 (70.2%)	1413 (61.9%)	3074 (74.9%)	1772 (63.4%)	2385 (73.7%)	330 (93.0%)
PAD, *n* (%)	1244 (19.5%)	171 (7.5%)	1073 (26.1%)	0 (0.0%)	3031 (93.6%)	301 (84.8%)
PVD, *n* (%)	1382 (21.6%)	219 (9.6%)	1163 (28.3%)	168 (6.0%)	1046 (32.3%)	168 (47.3%)
Prevalent anemia, *n* (%)	1371 (21.5%)	348 (15.2%)	1023 (24.9%)	494 (17.7%)	695 (21.5%)	182 (51.3%)
Prior cardiac procedure, *n* (%)	3318 (51.9%)	992 (43.4%)	2326 (56.7%)	1164 (41.6%)	1819 (56.2%)	335 (94.4%)
Proteinuria, *n* (%)	962 (15.1%)	410 (18.0%)	552 (13.4%)	362 (12.9%)	542 (16.7%)	58 (16.3%)
Pyelonephritis, *n* (%)	32 (0.5%)	10 (0.4%)	22 (0.5%)	8 (0.3%)	19 (0.6%)	5 (1.4%)
Resistant hypertension, *n* (%)	1229 (19.2%)	319 (14.0%)	910 (22.2%)	451 (16.1%)	663 (20.5%)	115 (32.4%)
Respiratory failure, *n* (%)	151 (2.4%)	32 (1.4%)	119 (2.9%)	27 (1.0%)	56 (1.7%)	68 (19.2%)
Sleep apnea, *n* (%)	1135 (17.8%)	428 (18.7%)	707 (17.2%)	431 (15.4%)	599 (18.5%)	105 (29.6%)
TIA, *n* (%)	142 (2.2%)	39 (1.7%)	103 (2.5%)	26 (0.9%)	80 (2.5%)	36 (10.1%)
**Lifestyle factors**						
Alcohol abuse, *n* (%)	74 (1.2%)	18 (0.8%)	56 (1.4%)	21 (0.8%)	42 (1.3%)	11 (3.1%)
Influenza vaccination, *n* (%)	1897 (29.7%)	948 (41.5%)	949 (23.1%)	823 (29.4%)	970 (30.0%)	104 (29.3%)
Smoking or nicotine dependence, *n* (%)	465 (7.3%)	172 (7.5%)	293 (7.1%)	161 (5.8%)	250 (7.7%)	54 (15.2%)
Smoking cessation, *n* (%)	274 (4.3%)	96 (4.2%)	178 (4.3%)	124 (4.4%)	132 (4.1%)	18 (5.1%)
**Laboratory value**						
HbA1c level						
Mean (SD)	9.2 (3.9)	9.2 (2.9)	9.2 (4.4)	8.9 (3.6)	9.4 (4.3)	9.1 (3.0)
Median [IQR]	8.4 [7.5, 9.8]	8.5 [7.5, 10.1]	8.4 [7.5, 9.8]	8.3 [7.4, 9.6]	8.6 [7.6, 10.1]	8.5 [7.4, 9.9]
Missing, *n* (%)	516 (8.1%)	178 (7.8%)	338 (8.2%)	236 (8.4%)	244 (7.5%)	36 (10.1%)
eGFR stage*						
G1, *n* (%)	754 (11.8%)	519 (22.7%)	235 (5.7%)	365 (13.0%)	360 (11.1%)	29 (8.2%)
G2, *n* (%)	1178 (18.4%)	468 (20.5%)	710 (17.3%)	511 (18.3%)	602 (18.6%)	65 (18.3%)
G3a, *n* (%)	2025 (31.7%)	590 (25.8%)	1435 (35.0%)	994 (35.5%)	903 (27.9%)	128 (36.1%)
G3b, *n* (%)	670 (10.5%)	118 (5.2%)	552 (13.4%)	275 (9.8%)	343 (10.6%)	52 (14.6%)
G4, *n* (%)	71 (1.1%)	9 (0.4%)	62 (1.5%)	26 (0.9%)	35 (1.1%)	10 (2.8%)
G5, *n* (%)	7 (0.1%)	4 (0.2%)	3 (0.1%)	2 (0.1%)	5 (0.2%)	0 (0.0%)
Missing, *n* (%)	1684 (26.4%)	576 (25.2%)	1108 (27.0%)	624 (22.3%)	989 (30.6%)	71 (20.0%)
UACR stage*						
A1, *n* (%)	794 (12.4%)	267 (11.7%)	527 (12.8%)	374 (13.4%)	384 (11.9%)	36 (10.1%)
A2, *n* (%)	2771 (43.4%)	1152 (50.4%)	1619 (39.4%)	1107 (39.6%)	1544 (47.7%)	120 (33.8%)
A3, *n* (%)	916 (14.3%)	323 (14.1%)	593 (14.4%)	350 (12.5%)	512 (15.8%)	54 (15.2%)
Missing, *n* (%)	1908 (29.9%)	542 (23.7%)	1366 (33.3%)	966 (34.5%)	797 (24.6%)	145 (40.8%)
Elevated hematocrit, *n* (%)	179 (2.8%)	76 (3.3%)	103 (2.5%)	72 (2.6%)	99 (3.1%)	8 (2.3%)
Rate of eGFR decline per year of follow-up						
For cardio-kidney events						
Mean (SD)	8.7 (13.6)	7.6 (10.9)	9.4 (15.1)	7.2 (11.9)	9.9 (15.2)	12.4 (13.1)
Median [IQR]	4.5 [2.1, 9.4]	4.4 [2.1, 9.0]	4.7 [2.1, 10.1]	4.2 [1.9, 7.8]	4.9 [2.6, 11.2]	6.7 [1.7, 18.3]
Missing, *n* (%)	5761 (90.2%)	2026 (88.7%)	3735 (91.0%)	2477 (88.6%)	2959 (91.4%)	325 (91.5%)
For treatment failure						
Mean (SD)	4.4 (6.8)	4.8 (6.3)	4.1 (7.2)	–	–	–
Median [IQR]	2.6 [1.3, 5.0]	2.8 [1.5, 5.2]	2.5 [1.2, 4.8]	–	–	–
Missing, *n* (%)	5337 (83.5%)	1865 (81.7%)	3472 (84.6%)	–	–	–

Abbreviations: *ACS* acute coronary syndrome, *CABG* coronary artery bypass grafting, *DKD* diabetic kidney disease, *COPD* chronic obstructive pulmonary disease, *CV* cardiovascular, *DSCI* diabetes severity and complications index, *GERD* gastroesophageal reflux disease, *IQR* interquartile range, *MC* microvascular complications, *NMSC* nonmelanoma skin cancer, *PAD* peripheral artery disease, *PTCA* percutaneous transluminal coronary angioplasty, *PVD* peripheral vascular disease, *SD* standard deviation, *SGLT2i* sodium-glucose co-transporter-2 inhibitor, *T2D* type 2 diabetes, *TIA* transient ischemic attack

Approximately 71.1% of patients in the total study population filled a metformin prescription in the year before SGLT2i initiation. The majority of the population filled at least one prescription for a second-line antidiabetic therapy in baseline (86.5%), while nearly half of the patients used at least two second-line antidiabetic therapies at baseline (46.5%). Approximately 82.0% of the total study population filled a statin prescription in the year before SGLT2i initiation. The most commonly filled antihypertensive medications were ACEi/ARBs (82.4%), followed by diuretics (54.2%), beta blockers (48%), and CCBs (39.9%). Percentages were approximately the same among subgroups (Table 3).

**Table 3:** Baseline use of medications among SGLT2i initiators with DKD, in the total study population and stratified by insurance type and CV risk

	SGLT2i initiators with DKD (***N*** = 6389)	Commercial insurance (***N*** = 2284)	Medicare insurance (***N*** = 4105)	Low CV risk (N = 2797)	Moderate CV risk (***N*** = 3237)	High CV risk (***N*** = 355)
**T2D medication**						
Metformin, *n* (%)	4545 (71.1%)	1739 (76.1%)	2806 (68.4%)	2063 (73.8%)	2256 (69.7%)	226 (63.7%)
Any second LOT, *n* (%)	5528 (86.5%)	1961 (85.9%)	3567 (86.9%)	2385 (85.3%)	2834 (87.6%)	309 (87.0%)
SU, *n* (%)	3093 (48.4%)	1004 (44.0%)	2089 (50.9%)	1387 (49.6%)	1555 (48.0%)	151 (42.5%)
TZD, *n* (%)	848 (13.3%)	273 (12.0%)	575 (14.0%)	371 (13.3%)	436 (13.5%)	41 (11.5%)
DPP4i, *n* (%)	2201 (34.4%)	785 (34.4%)	1416 (34.5%)	1024 (36.6%)	1074 (33.2%)	103 (29.0%)
GLP1ra, *n* (%)	1385 (21.7%)	680 (29.8%)	705 (17.2%)	551 (19.7%)	763 (23.6%)	71 (20.0%)
Basal insulin, *n* (%)	2249 (35.2%)	801 (35.1%)	1448 (35.3%)	812 (29.0%)	1258 (38.9%)	179 (50.4%)
AGI, *n* (%)	58 (0.9%)	13 (0.6%)	45 (1.1%)	22 (0.8%)	33 (1.0%)	3 (0.8%)
Meglitinide, *n* (%)	170 (2.7%)	55 (2.4%)	115 (2.8%)	84 (3.0%)	73 (2.3%)	13 (3.7%)
Any two second LOTs, *n* (%)	2974 (46.5%)	1042 (45.6%)	1932 (47.1%)	1222 (43.7%)	1588 (49.1%)	164 (46.2%)
SU + TZD, *n* (%)	391 (6.1%)	122 (5.3%)	269 (6.6%)	180 (6.4%)	199 (6.1%)	12 (3.4%)
SU + DPP4i, *n* (%)	1010 (15.8%)	342 (15.0%)	668 (16.3%)	490 (17.5%)	488 (15.1%)	32 (9.0%)
SU + GLP1ra, *n* (%)	377 (5.9%)	183 (8.0%)	194 (4.7%)	164 (5.9%)	199 (6.1%)	14 (3.9%)
SU + basal insulin, *n* (%)	569 (8.9%)	185 (8.1%)	384 (9.4%)	224 (8.0%)	307 (9.5%)	38 (10.7%)
TZD + DPP4i, *n* (%)	249 (3.9%)	88 (3.9%)	161 (3.9%)	124 (4.4%)	116 (3.6%)	9 (2.5%)
TZD + GLP1ra, *n* (%)	118 (1.8%)	55 (2.4%)	63 (1.5%)	53 (1.9%)	57 (1.8%)	8 (2.3%)
TZD + basal insulin, *n* (%)	152 (2.4%)	54 (2.4%)	98 (2.4%)	60 (2.1%)	84 (2.6%)	8 (2.3%)
DPP4i + basal insulin, *n* (%)	430 (6.7%)	142 (6.2%)	288 (7.0%)	157 (5.6%)	239 (7.4%)	34 (9.6%)
GLP1ra + basal insulin, *n* (%)	483 (7.6%)	233 (10.2%)	250 (6.1%)	166 (5.9%)	291 (9.0%)	26 (7.3%)
Basal + mealtime insulin, *n* (%)	826 (12.9%)	253 (11.1%)	573 (14.0%)	276 (9.9%)	478 (14.8%)	72 (20.3%)
**CV medication**						
Alpha blocker, *n* (%)	194 (3.0%)	45 (2.0%)	149 (3.6%)	80 (2.9%)	99 (3.1%)	15 (4.2%)
ACEi/ARB, *n* (%)	5263 (82.4%)	1831 (80.2%)	3432 (83.6%)	2282 (81.6%)	2682 (82.9%)	299 (84.2%)
DRI, *n* (%)	7 (0.1%)	0 (0.0%)	7 (0.2%)	4 (0.1%)	3 (0.1%)	0 (0.0%)
Antiplatelet, *n* (%)	957 (15.0%)	268 (11.7%)	689 (16.8%)	228 (8.2%)	560 (17.3%)	169 (47.6%)
Anticoagulant, *n* (%)	513 (8.0%)	117 (5.1%)	396 (9.6%)	188 (6.7%)	251 (7.8%)	74 (20.8%)
Aspirin, *n* (%)	99 (1.5%)	88 (3.9%)	11 (0.3%)	31 (1.1%)	53 (1.6%)	15 (4.2%)
Beta blocker, *n* (%)	3065 (48.0%)	838 (36.7%)	2227 (54.3%)	1160 (41.5%)	1637 (50.6%)	268 (75.5%)
CCB, *n* (%)	2548 (39.9%)	748 (32.7%)	1800 (43.8%)	1059 (37.9%)	1328 (41.0%)	161 (45.4%)
Central alpha agonist, *n* (%)	298 (4.7%)	87 (3.8%)	211 (5.1%)	113 (4.0%)	162 (5.0%)	23 (6.5%)
Diuretic, *n* (%)	3462 (54.2%)	1068 (46.8%)	2394 (58.3%)	1443 (51.6%)	1771 (54.7%)	248 (69.9%)
ENaC, *n* (%)	179 (2.8%)	63 (2.8%)	116 (2.8%)	92 (3.3%)	80 (2.5%)	7 (2.0%)
Loop diuretic, *n* (%)	1248 (19.5%)	274 (12.0%)	974 (23.7%)	397 (14.2%)	687 (21.2%)	164 (46.2%)
MRA, *n* (%)	339 (5.3%)	96 (4.2%)	243 (5.9%)	104 (3.7%)	186 (5.7%)	49 (13.8%)
K+ sparing diuretic, *n* (%)	507 (7.9%)	155 (6.8%)	352 (8.6%)	193 (6.9%)	259 (8.0%)	55 (15.5%)
Thiazide diuretic, *n* (%)	2547 (39.9%)	872 (38.2%)	1675 (40.8%)	1149 (41.1%)	1262 (39.0%)	136 (38.3%)
Digoxin, *n* (%)	143 (2.2%)	31 (1.4%)	112 (2.7%)	50 (1.8%)	75 (2.3%)	18 (5.1%)
Statin, *n* (%)	5236 (82.0%)	1775 (77.7%)	3461 (84.3%)	2225 (79.5%)	2695 (83.3%)	316 (89.0%)
Nitrate, *n* (%)	539 (8.4%)	103 (4.5%)	436 (10.6%)	128 (4.6%)	307 (9.5%)	104 (29.3%)
Oral anticoagulant, *n* (%)	484 (7.6%)	107 (4.7%)	377 (9.2%)	176 (6.3%)	244 (7.5%)	64 (18.0%)
K+ binding agent, *n* (%)	42 (0.7%)	10 (0.4%)	32 (0.8%)	8 (0.3%)	28 (0.9%)	6 (1.7%)
K+ supplement, *n* (%)	608 (9.5%)	153 (6.7%)	455 (11.1%)	202 (7.2%)	328 (10.1%)	78 (22.0%)

Abbreviations: *AGI* alpha glucosidase inhibitor, *DKD* diabetic kidney disease, *CV* cardiovascular, *DPP4i* dipeptidyl peptidase-4 inhibitor, *GLP1ra* glucagon-like peptide-1 receptor agonist, *LOT* line of therapy, *SU* sulfonylurea, *TZD* thiazolidinedione, *ACEi* angiotensin-converting enzyme inhibitor, *ARB* angiotensin receptor blocker, *CCB* calcium channel blocker, *CKD* chronic kidney disease, *CV* cardiovascular, *DRI* direct renin inhibitor, *eGFR* estimated glomerular filtration rate, *ENaC* epithelial sodium channel blocker, *K+* potassium, *MRA* mineralocorticoid receptor antagonist, *SGLT2i* sodium-glucose co-transporter-2 inhibitor, *T2D* type 2 diabetes

### Cardio-kidney events

**Fig. 3:**
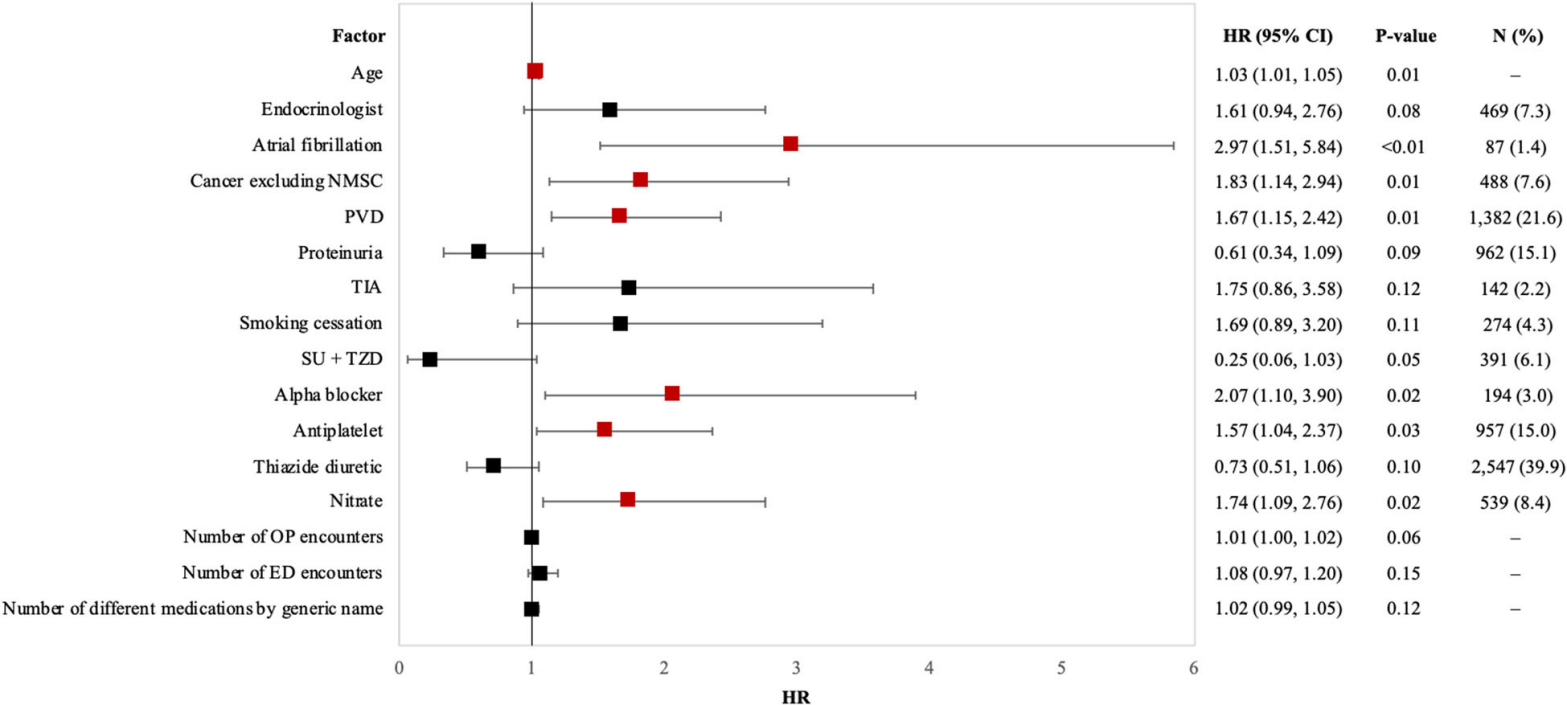
Multivariate HRs (95% CIs) for factors associated with CV hospitalization among initiators of SGLT2i with DKD (*N* = 6389). Statistically significant (*p* value < 0.05) factors associated with a positive effect are in red and a negative effect are in green. Abbreviations: CI = confidence interval, ED = emergency department, HR = hazard ratio, OP = outpatient, PVD = peripheral vascular disease, SU = sulfonylurea, TIA = transient ischemic attack, TZD = thiazolidinedione

The rate of CV hospitalization was 26.0 (95% CI 21.6, 30.4) events per 1000 PY in the overall population assessed over a mean follow-up time of 292.3 (SD 328.5) days; the highest being in the high CV risk subgroup 79.5 (95% CI 40.5, 118.4) and in Medicare patients [32.9 (95% CI 26.4, 39.4), Table 4]. Baseline characteristics associated with higher risk of CV hospitalization included age [multivariate hazard ratio (HR) of 1.03 (95% CI 1.01, 1.05) per year of increased age], atrial fibrillation [HR 2.97 (CI 1.51, 5.84)], peripheral vascular disease (PVD) [HR 1.67 (CI 1.15, 2.42)], and cancer excluding nonmelanoma skin cancer (NMSC) [HR 1.83 (CI 1.14, 2.94)]. The risk of CV hospitalization also increased significantly with the baseline use of alpha blockers [HR 2.07 (CI 1.10, 3.90)], antiplatelets [HR 1.57 (CI 1.04, 2.37)], and nitrates [HR 1.74 (CI 1.09, 2.76), Fig. 3].

**Table 4:** Observed cardio-kidney or treatment failure-related outcomes among patients with DKD initiating SGLT2i

	SGLT2i initiators with DKD (***N*** = 6389)	Commercial insurance (***N*** = 2284)	Medicare insurance (***N*** = 4105)	Low CV risk (***N*** = 2797)	Moderate CV risk (***N*** = 3237)	High CV risk (***N*** = 355)
**CV hospitalization**						
Mean (SD) follow-up time in days	292.3 (328.5)	336.3 (368.0)	267.8 (301.5)	333.0 (376.0)	266.4 (284.8)	207.0 (249.7)
Rate per 1000 PY (95% CI)	26.0 (21.6, 30.4)	16.2 (10.7, 21.6)	32.9 (26.4, 39.4)	16.5 (11.5, 21.4)	31.7 (24.6, 38.9)	79.5 (40.5, 118.4)
**Kidney hospitalization**						
Mean (SD) follow-up time in days	294.6 (329.8)	339.1 (369.4)	269.9 (302.9)			
Rate per 1000 PY (95% CI)	12.0 (9.0, 15.0)	5.7 (2.5, 8.9)	16.5 (11.9, 21.0)			
**AKI hospitalization**						
Mean (SD) follow-up time in days	293.5 (330.0)	338.9 (370.6)	268.3 (302.2)			
Rate per 1000 PY (95% CI)	22.8 (18.7, 26.9)	12.3 (7.6, 17.0)	30.2 (24.0, 36.4)			
**Discontinuation of SGLT2is**						
Discontinuation, *n* (%)	3512 (55.0%)	1024 (44.8%)	2488 (60.6%)			
Mean (SD) time to discontinuation in days	300.6 (243.9)	331.4 (282.5)	287.9 (225.0)			
Median [IQR] time to discontinuation in days	204 [144, 364]	221 [143, 420]	195 [145, 348]			
**Treatment failure**						
Mean (SD) follow-up time in days	361.49 (365.96)	347.14 (371.94)	369.47 (362.39)			
Rate per 1000 PY (95% CI)	510.46 (492.86, 528.07)	514.21 (484.06, 544.37)	508.51 (486.82, 530.19)			
**Reason for treatment failure**						
SGLT2i discontinuation or switch from SGLT2i to another antidiabetic drug class, *n* (%)	3053 (63.6%)					
Addition of another antidiabetic class not used in baseline, *n* (%)	1495 (31.1%)					
Initiation of insulin, *n* (%)	213 (4.4%)					
Two or more reasons for treatment failure, *n* (%)	41 (0.9%)					

Abbreviations: *AKI* acute kidney injury, *DKD* diabetic kidney disease, *CI* confidence interval, *CV* cardiovascular, *PY* person-years, *SD* standard deviation, *SGLT2i* sodium-glucose co-transporter-2 inhibitor, *T2D* type 2 diabetes

**Fig. 4:**
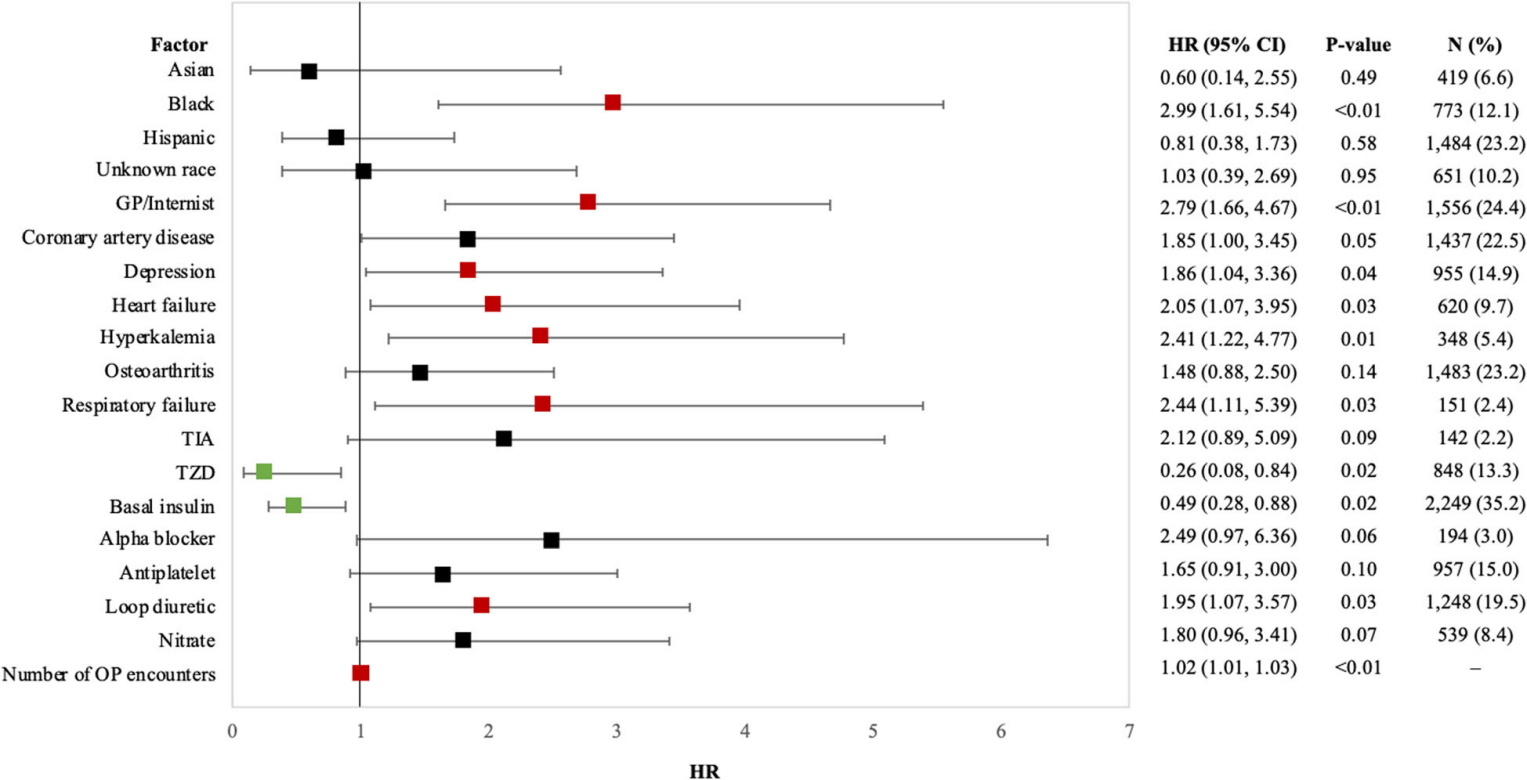
Multivariate HRs (95% CIs) for factors associated with kidney hospitalization among initiators of SGLT2i with DKD (*N* = 6389). * Statistically significant (*p* value < 0.05) factors associated with a positive effect are in red and a negative effect are in green. Abbreviations: CI = confidence interval, ED = emergency department, HR = hazard ratio, OP = outpatient, PVD = peripheral vascular disease, SU = sulfonylurea, TIA = transient ischemic attack, TZD = thiazolidinedione

The rate of kidney hospitalization was 12.0 (95% CI 9.0, 15.0) events per 1000 PY, evaluated over a mean follow-up time of 294.6 (SD 329.8) days. Baseline characteristics with higher risk of kidney hospitalization included Black race [HR 2.99 (CI 1.61, 5.54)], the provider being a general practitioner or internist [HR 2.79 (CI 1.66, 4.67)], and the number of outpatient encounters [HR 1.02 (CI 1.01, 1.03) per additional encounter]. The risk also increased significantly with baseline evidence of heart failure, hyperkalemia, respiratory failure, and depression, as well as the baseline use of loop diuretics (Fig. 4).

**Fig. 5:**
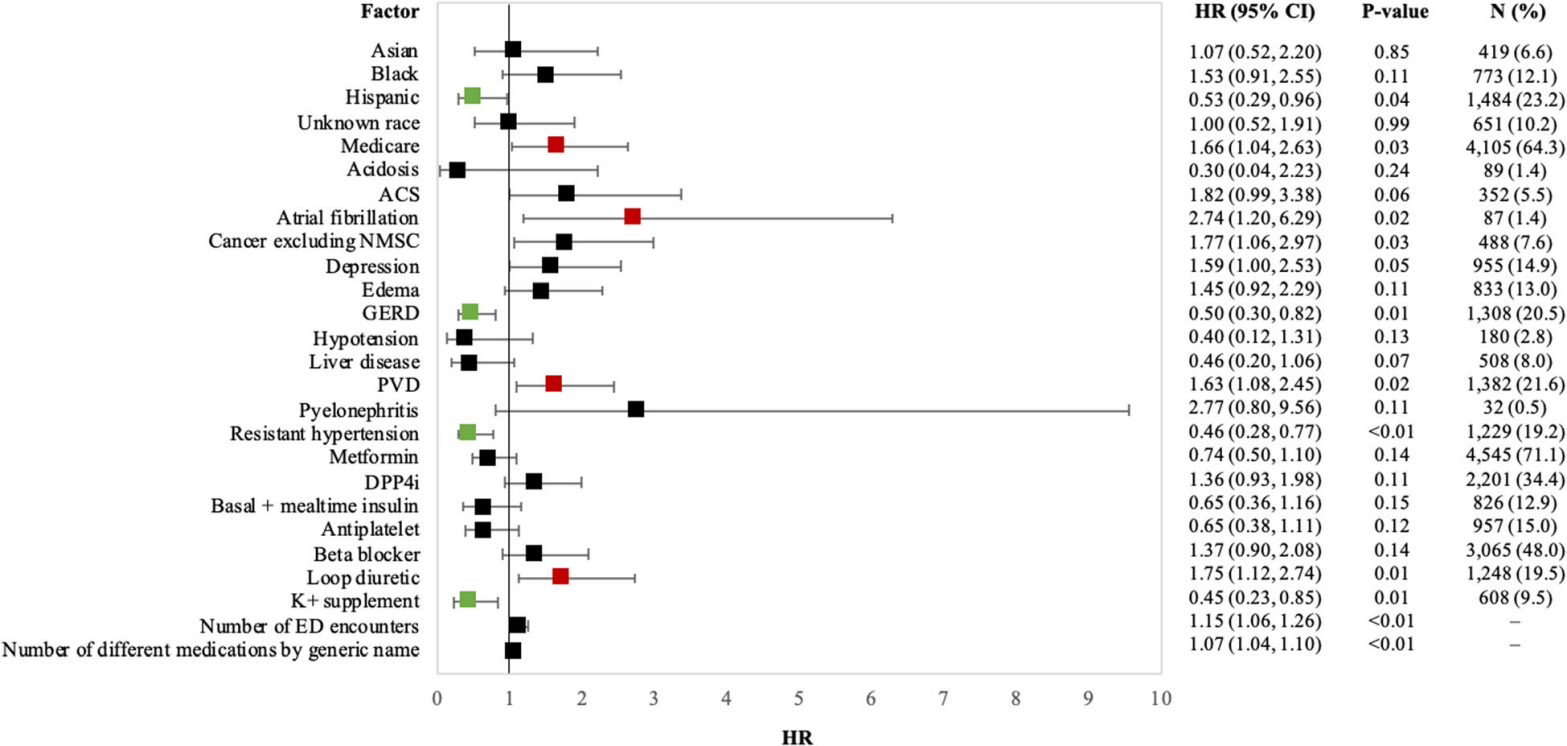
Multivariate HRs (95% CIs) for factors associated with AKI hospitalization among initiators of SGLT2i with DKD (*N* = 6389). * Statistically significant (*p* value < 0.05) factors associated with a positive effect are in red and a negative effect are in green. Abbreviations: ACS = acute coronary syndrome, CI = confidence interval, DPP4i = dipeptidyl peptidase-4 inhibitor, ED = emergency department, GERD = gastroesophageal reflux disease, HR = hazard ratio, K+ = potassium, NMSC = nonmelanoma skin cancer, PVD = peripheral vascular disease

The rate of AKI hospitalization was 22.8 (95% CI 18.7, 26.9) events per 1000 PY, captured over a mean follow-up time of 293.5 (SD 330.0) days. AKI hospitalization risk significantly increased with being a Medicare beneficiary [HR 1.66 (CI 1.04, 2.63)], having baseline evidence of atrial fibrillation [HR 2.74 (CI 1.20, 6.29)], PVD [HR 1.63 (CI 1.08, 2.45)], or cancer excluding NMSC [HR 1.77 (CI 1.06, 2.97)], and the use of loop acting diuretics [HR 1.75 (CI 1.12, 2.74), Fig. 5].

### Treatment failure

Among the total population, 55.0% of patients overall, and 44.8% and 60% in the commercially insured and Medicare subgroups, respectively, discontinued treatment (90-day grace period) during the follow-up period. The median time to discontinuation of SGLT2is was 204 [IQR 144, 364] days.

The rate of treatment failure was 510.5 (95% CI 492.9, 528.1) events per 1000 PY over an average of 361.5 (SD 366.0) days of follow-up. The reason for treatment failure in more than half (63.6%) of all treatment failure events in follow-up (*N* = 4802) was attributed to SGLT2i discontinuation or a switch from SGLT2is to another antidiabetic drug class. One-third (31.1%) of treatment failure events were due to the addition of another antidiabetic drug class to therapy, which was not previously used in baseline (Table 4).

**Fig. 6:**
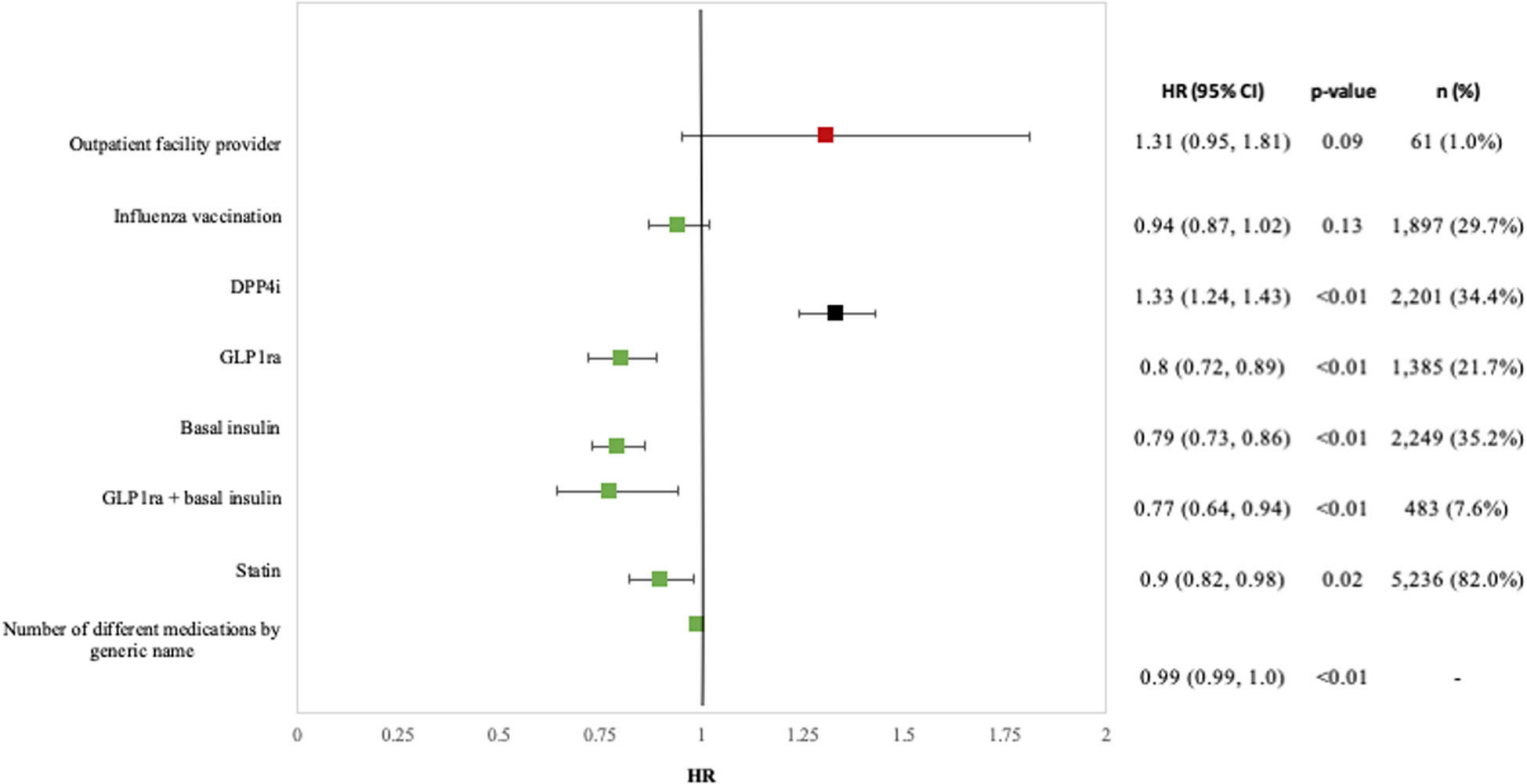
Multivariate HRs (95% CIs) for factors associated with treatment failure among initiators of SGLT2i with DKD (*N* = 6389). * Statistically significant (*p* value < 0.05) factors associated with a positive effect are in red and a negative effect are in green. Abbreviations: CI = confidence interval, DPP4i = dipeptidyl peptidase-4 inhibitor, GLP1ra = glucagon-like peptide-1 receptor agonist, HR = hazard ratio. Combined abbreviations for Additional Files: DPP4i = dipeptidyl peptidase-4 inhibitor, GLP1ra = glucagon-like peptide-1 receptor agonist, DKD = diabetic kidney disease, SGLT2i = sodium-glucose co-transporter-2 inhibitor, T2D = type 2 diabetes, CV = cardiovascular, CVD = Cardiovascular disease, ACEi = angiotensin-converting enzyme inhibitor, AKI = acute kidney injury, eGFR = Estimated glomerular filtration rate, ESKD = end-stage kidney disease, HbA1c = hemoglobin A1c, ICD = International Classifications of Diseases, NMSC = nonmelanoma skin cancer, UACR = urine albumin-to-creatinine ratio, NDC = National Drug Code

The risk of treatment failure increased significantly with the baseline use of DPP4is [HR 1.33 (CI 1.24, 1.43)] and non-significantly with outpatient facility providers [HR 1.31 (CI 0.95, 1.81)]. In contrast, the risk of treatment failure was significantly decreased with baseline use of statins [HR 0.9 (CI 0.82, 0.98)], GLP1ra [HR 0.8 (CI 0.72, 0.89)], basal insulin [HR 0.79 (CI 0.73, 0.86)], or concurrent use of both [HR 0.77 (CI 0.64, 0.94)] (Fig. 6).

Results from the sensitivity analysis of key time-dependent SGLT2i adverse events (Additional file 1: Table S2) showed that, overall, a patient experiencing diabetic ketoacidosis during follow-up was significantly associated with risk of treatment failure [(HR 2.43 (95% CI 1.17, 5.07)] and commercially insured patients had similar results to the overall population. In older Medicare patients, volume depletion was associated with higher risk of treatment failure (Additional file 1: Table S2).

## Discussion

In this US-based cohort study determining factors associated with cardio-kidney complications and treatment failure after the initiation of SGLT2is among DKD patients, we observed high residual risks of cardio-kidney events, especially in patients with high cardiovascular risk, with certain comorbidities and those on Medicare insurance plans, and high rates of treatment failure, potentially due to adverse events (AE) related to SGLT2is.

Among our study population, very few patients were at a late stage of CKD (stages G4 or G5), while a high proportion of patients had mild to moderate decreased eGFR (CKD stage G3) at the time of SGLT2i initiation, conforming with indications during the study period for this drug class. During follow-up, we observed high cardio-kidney events rates, particularly in patients with moderate to high CV risk, or prior CV comorbidities, such as atrial fibrillation and PVD; or patients with evidence of frequently reported side effect, such as hyperkalemia, or depression [21, 22]. Additionally, we observed high rates of treatment discontinuation and treatment switching from SGLT2is, which was significantly associated with the development of potential SGLT2i adverse events.

Our results reflect physician adherence with current drug recommendations for SGLT2i initiation in kidney impaired T2D patients [10]. SGLT2is were found to have protective cardio-kidney effects in several clinical trials and observational studies [23, 24]. However, our findings indicate the presence of residual risk of cardio-kidney outcomes after SGLT2i initiation among patients with DKD, particularly in patients with high CV risk or prior cardio-kidney events, similarly to what was described in clinical trials that found slightly higher event rates of cardio-kidney outcomes in established CVD patients [11, 13].

The discontinuation rates found in our study population were slightly higher than existing research on discontinuation of SGLT2i among T2D patients [14, 25], which could be explained by the development of SGLT2i adverse events. These events were higher among patients with treatment failure compared to their counterparts during follow-up (Additional file 1: Table S3), specifically diabetic ketoacidosis among commercially insured patients and volume depletion among the older Medicare patients, although the percentages of patients with these adverse event during follow-up was in line with the percentages at baseline in the overall population. Therefore, further research is needed to assess potential relationships between adverse events and treatment discontinuation.

Ultimately, the residual risk, combined with the observed high treatment discontinuation rates of SGLT2is, reveals suboptimal treatment in a significant proportion of this vulnerable population and suggests the need for additional cardio- and renoprotective interventions [26]. The clinical profiles of patients with high unmet needs must be further explored to achieve the best clinical outcomes.

### Limitations

Our study had several limitations. First, administrative claims data carries a potential for misclassification of patients’ diagnoses, since the presence of a diagnosis code may not indicate the presence of a disease, but a rule out code in some instances. To address this limitation in identifying DKD, validated algorithms for identification of T2D patients from inpatient, outpatient, and prescription claims, as well as two laboratory values indicating kidney disease to identify CKD patients, were used. Nonetheless, DKD can only be accurately defined by histopathologic screening of the kidney [27]. Second, not all important comorbidities or risk factors can be accurately ascertained within claims data, such as a patient’s smoking status, body mass index, and other CKD symptoms (e.g., poor appetite, weakness, or itching). As a result, we may not be capturing all relevant factors associated with the outcomes of interest. Third, laboratory results in the database were incomplete since laboratory orders were not always represented in claims. In addition, laboratory results that were done in a hospital setting or directly in the physician office were not represented in the database. While there is no reason to suspect a selection bias as to which patients receive laboratory results, some laboratory results may be missing and so some patients may have been misclassified as incident CKD cases due to unobservable prior lab results that indicate kidney disease. Fourth, dispensing data only covers claims filed for insurance coverage. Generic varieties for drugs (e.g., metformin) are often available and may be affordable enough so that patients never file claims for them. As a result, these dispensings were not captured in this study. Pauly et al. estimate that about 30% of metformin and ACEi fills are done through low-cost generic programs [28]. Our results, therefore, might have slightly underestimated the real use of such drugs during the baseline period, the switching or addition of generic drugs during follow-up and potentially limited the possibility to completely capture reasons for discontinuation. Additionally, therapeutic inertia, although out of the scope of our study population, is an additional reason that could deprive patients of beneficial medications [29]. Fifth, the cardio-kidney outcomes rates estimated in our study might be underestimated, since the follow-up was allowed to be censored on SGLT2i discontinuation; therefore, the risk of occurrence of these outcomes is not included in the estimation (as it would be in a intention-to-treat approach). Lastly, in addition to the studied factors, there might be other characteristics that could predict the studied outcomes. However, based on our study design, we can only draw conclusions of association, and not causation, between the studied factors and the outcomes.

## Conclusion

Our study demonstrated a high unmet need with high rates of cardio-kidney outcomes among patients with DKD treated with SGLT2is in clinical practice and high rates of SGLT2i treatment failure. Patients with high baseline CV risk and the presence of certain conditions, such as atrial fibrillation, PVD, and heart failure, were at higher risk for cardio-kidney events and might particularly benefit from further treatment options. Further research is needed to assess the potential relationship between adverse events and SGLT2i treatment failure.

## Supplementary Information

**Additional file 1: Table S1.** Definitions of all variables used or explored in this study. Table S2: Overall Multivariate Time Dependent Assessment of Factors Associated with Treatment Failure among SGLT2i initiators with DKD and stratified by insurance. Table S3: Adverse Events of Interest over Follow-up

**Additional file 2: Figure S1.** CV Hospitalization outcome among subgroups. **Figure S2.** Renal Hospitalization among subgroups. **Figure S3.** AKI Hospitalization among subgroups. **Figure S4.** Treatment Failure among subgroups

## Data Availability

The datasets generated and/or analyzed for this study are not publicly available because the data source is owned by a third-party (Optum™ Clinformatics™ Data Mart). Bayer AG has a license for analysis of data from this source. As such, the authors cannot provide the raw data themselves. Other researchers can access the data by purchase through Optum™.

## Abbreviations

ACEi: Angiotensin-converting enzyme inhibitor
AE: Adverse event
AKI: Acute kidney injury
CDM: Clinformatics Data Mart
CI: Confidence intervals
CKD: Chronic kidney disease
CV: Cardiovascular
CVD: Cardiovascular disease
DKD: Diabetic kidney disease
DPP4i: Dipeptidyl peptidase-4 inhibitors
eGFR: Estimated glomerular filtration rate
ESKD: End-stage kidney disease
GLP1ra: Glucagon-like peptide-1 receptor agonists
HbA1c: Hemoglobin A1c
HR: Hazard ratio
ICD: International Classifications of Diseases
IQR: Interquartile range
NMSC: Nonmelanoma skin cancer
PY: Person-years
SD: Standard deviation
SGLT2i: Sodium-glucose co-transporter 2 inhibitor
T2D: Type 2 diabetes
UACR: Urine albumin-to-creatinine ratio
US: United States
