# Supplementary material for: Predictors of cardio-kidney complications and treatment failure in patients with chronic kidney disease and type 2 diabetes treated with SGLT2 inhibitors
Source: BMC Med. 2022 Jan 10;20:2. doi: 10.1186/s12916-021-02191-2 (PMC8744296; doi:10.1186/s12916-021-02191-2)
Supplement: Supplementary file 1 — Additional file 1: Table S1. Definitions of all variables used or explored in this study. Table S2: Overall Multivariate Time Dependent Assessment of Factors Associated with Treatment Failure among SGLT2i initiators with DKD and stratified by insurance. Table S3: Adverse Events of Interest over Follow-up [file 12916_2021_2191_MOESM1_ESM.docx]

[Additional File 1: Table 1. Definitions of all variables used or explored in this study. 2](#_Toc87958073)

[Additional File 1: Table 2: Overall Multivariate Time Dependent Assessment of Factors Associated with Treatment Failure among SGLT2i initiators with DKD and stratified by insurance 20](#_Toc87958074)

[Additional File 1: Table 3: Adverse Events of Interest over Follow-up 22](#_Toc87958075)

# **Additional File 1: Table 1. Definitions of all variables used or explored in this study.**

| **Category** | **Variable** | **Categorization/Definition** |
| --- | --- | --- |
| Selection Criteria | SGLT2i | One pharmacy claim with generic NDC code for canagliflozin, dapagliflozin propanediol, empagliflozin, canagliflozin/metformin HCL, dapagliflozin propanediol/metformin HCL,  empagliflozin/metformin HCL, dapagliflozin  propanediol/saxagliptin HCL, or empagliflozin/linagliptin |
| Selection Criteria | Age | Continuous variable of age in years |
| Selection Criteria | Type 2 diabetes diagnosis | One of the following:  ● One inpatient claim for ICD-9 diagnosis code 250.40, 250.42, 250.00, 250.02, 250.10, 250.12, 250.20, 250.22, 250.30, 250.32, 250.50, 250.52, 250.60, 250.62, 250.70, 250.72, 250.80, 250.82, 250.90, 250.92 (any position) or ICD-10 diagnosis code E11.2, E11.21, E11.22, E11.29, E11.0, E11.00, E11.01, E11.3, E11.31, E11.311, E11.319, E11.32, E11.321, E11.329, E11.33, E11.331, E11.339, E11.34, E11.341, E11.349, E11.35, E11.351, E11.359, E11.36, E11.39, E11.4, E11.40, E11.41, E11.42, E11.43, E11.44, E11.49, E11.5, E11.51, E11.52, E11.59, E11.6, E11.61, E11.610, E11.618, E11.62, E11.620, E11.621, E11.622, E11.628, E11.63, E11.630, E11.638, E11.64, E11.641, E11.649, E11.65, E11.69, E11.8, E11.9 (any position); and/or  ● Two outpatient claims for ICD 9 diagnosis code 250.40, 250.42, 250.00, 250.02, 250.10, 250.12, 250.20, 250.22, 250.30, 250.32, 250.50, 250.52, 250.60, 250.62, 250.70, 250.72, 250.80, 250.82, 250.90, 250.92 (any position) or ICD-10 diagnosis code E11.2, E11.21, E11.22, E11.29,  E11.0, E11.00, E11.01, E11.3, E11.31, E11.311, E11.319, E11.32, E11.321, E11.329, E11.33, E11.331, E11.339, E11.34, E11.341, E11.349, E11.35, E11.351, E11.359, E11.36, E11.39, E11.4, E11.40, E11.41, E11.42, E11.43, E11.44, E11.49, E11.5, E11.51, E11.52, E11.59, E11.6, E11.61, E11.610, E11.618, E11.62, E11.620, E11.621, E11.622, E11.628, E11.63, E11.630, E11.638, E11.64, E11.641, E11.649, E11.65, E11.69, E11.8, E11.9 (any position) at least 30 days apart but no more than 365 days apart; and/or  ● One pharmacy claim with NDC generic name for acarbose, sitagliptin phosphate, sitagliptin phosphate/simvastatin, saxagliptin HCL, linagliptin, alogliptin benzoate, alogliptin benzoate/pioglitazone HCL, exenatide, exenatide microspheres, liraglutide, albiglutide, dulaglutide, lixisenatide, semaglutide, insulin degludec/liraglutide, insulin glargine,human recombinant analog/lixisenatide, nateglinide, repaglinide, repaglinide-metformin HCL, miglitol, colesevelam HCL, canagliflozin, dapagliflozin propanediol, empagliflozin, dapagliflozin, propanediol/saxagliptin HCL, empagliflozin/linagliptin, chlorpropamide, glimepiride, glipizide, glyburide, glyburide,micronized, pioglitazone HCL/glimepiride, rosiglitazone maleate/glimepiride, pioglitazone HCL, rosiglitazone maleate, rosiglitazone maleate/glimepiride, pioglitazone HCL/glimepiride, alogliptin, benzoate/pioglitazone HCL, sitagliptin, phosphate/metformin HCL, saxagliptin HCL/metformin HCL, linagliptin/metformin HCL, alogliptin, benzoate/metformin HCL, canagliflozin/metformin HCL, empagliflozin/metformin HCL, dapagliflozin propanediol/metformin HCL, glipizide/metformin HCL, glyburide/metformin HCL, glipizide/metformin HCL, glyburide/metformin HCL, rosiglitazone, maleate/metformin HCL, pioglitazone HCL/metformin HCL, insulin aspart, insulin aspart protamine human/insulin aspart, insulin detemir, insulin glargine,human recombinant analog, insulin glulisine, insulin isophane nph,bf-pk, insulin isophane,beef, insulin isophane,beef pure, insulin isophane,pork pure, insulin lispro, insulin lispro protamine and insulin lispro, insulin nph human isophane, insulin nph human isophane/insulin regular, human, insulin nph human semi-syn, insulin nph human semi-syn/insulin reg human semi-syn, insulin protamine zinc,beef, insulin protamine zinc,beef (p), insulin protamine zinc,bf-pk, insulin reg human semi-syn, insulin reg, hum s-s buff, insulin regular, human, insulin regular, human/insulin release unit, insulin regular, human/insulin release unit/chamber/inhaler, insulin regular,beef-pork, insulin regular,human buffered, insulin zinc beef, insulin zinc extended human recombinant, insulin zinc extended,bf-pk, insulin zinc human recombinant, insulin zinc human semi-syn, insulin zinc prompt,bf-pk, insulin zinc,beef purified, insulin zinc,beef purified/insulin zinc,pork purified, insulin zinc,beef-pork, insulin zinc,pork purified, insulin,pork, insulin,pork purified, insulin,pork purified/insulin isophane,pork pure, insulin,pork reg. concentrate, insulin,beef, insulin degludec, diluent,insulin aspart combination no.1, insulin protamine zinc,pork (p), insulin pump cartridge, insulin pump controller, insulin pump syringe, 1.8 ml, insulin pump syringe, 3 ml, insulin pump/infusion set/blood-glucose meter, insulin zinc ext,beef (p), insulin zinc extended,beef, insulin zinc prompt,beef, insulin zinc prompt,pork pure, sub-q insulin delivery device, 20 unit,disposable, sub-q insulin delivery device, 30 unit, disposable, sub-q insulin delivery device, 40 unit, disposable, sub-q insulin pump, continuous glucose monitoring system, subcutaneous bolus insulin patch pump, 200 unit, disposable, or subcutaneous insulin pump |
| Selection Criteria | eGFR laboratory order | LOINC code is any of { 48642-3, 48643-1 } |
| Selection Criteria | UACR laboratory order | LOINC code is any of { 9318-7, 14959-1, 30000-4, 14958-3, 32294-1} |
| Selection Criteria | HIV/AIDS | One claim for ICD-9 inpatient or outpatient diagnosis code 042.xx 0.44.xx (any position) or ICD-10 diagnosis code inpatient or outpatient B20.x–B22.x, B24.x (any position) (Quan et al, 2005) |
| Selection Criteria | Sickle cell disease | One claim for ICD-9 inpatient or outpatient diagnosis code 282.6, 282.60, 282.61, 282.62, 282.63, or 282.69 (any position) or ICD-10 inpatient or outpatient diagnosis code D57.00, D57.01, D57.02, D57.20, D57.211, D57.212, D57.219, D57.40, D57.411, D57.412, D57.419, D57.80, D57.811, or D57.819 (any position) (Shankar et al, 2005; Singh et al, 2018) |
| Selection Criteria | Nephrotic syndrome,  including: focal  glomerulosclerosis/focal sclerosing glomerulonephritis, membranoproliferative  glomerulonephritis type 1/ diffuse  membranoproliferative  glomerulosclerosis | One claim for inpatient or outpatient ICD-9 diagnosis code 581.xx (any position) or ICD-10 diagnosis code N04 or N04.xx (any position) |
| Selection Criteria | Chronic glomerulonephritis, nephritis, nephritic Syndrome, and nephropathy, including: membranous nephropathy, IgA nephropathy/Berger’s disease, rapidly progressive, systemic lupus erythematosus nephritis, interstitial nephritis/pyelonephritis from analgesic abuse, chronic/acute interstitial nephritis | One claim for inpatient or outpatient ICD-9 diagnosis code 582.xx or 583.xx (any position) or ICD-10 diagnosis code N03.xx or N07.xx (any position) |
| Selection Criteria | Polyarteritis nodosa and allied conditions (Wegener’s granulomatosis) | One claim for inpatient or outpatient ICD-9 diagnosis code 446.xx (any position) or ICD-10 diagnosis code M30, M30.0, or M30.8 (any position) |
| Selection Criteria | Obstructive and reflux uropathy or urolithiasis | One claim for inpatient or outpatient ICD-9 diagnosis code 592.9, 599.6x (any position) or ICD-10 diagnosis code N20.9, N13.xx (any position) |
| Selection Criteria | Chronic pyelonephritis/reflux nephropathy | One claim for inpatient or outpatient ICD-9 diagnosis code 590.0xx 593.7x (any position) or ICD-10 diagnosis code N11.1, N13.729 (any position) |
| Selection Criteria | Renal artery occlusion or renal artery stenosis | One claim for inpatient or outpatient ICD-9 diagnosis code 362.32, 440.1 (any position) or ICD-10 diagnosis code I70.1, H34.239 (any position) |
| Selection Criteria | Polycystic kidneys (adult type) | One claim for inpatient or outpatient ICD-9 diagnosis code 753.1x (any position) or ICD-10 diagnosis code Q61.2 (any position) |
| Selection Criteria | Renal tumor (malignant) | One claim for inpatient or outpatient ICD-9 diagnosis code 189.0 (any position) or ICD-10 diagnosis code C64.9 (any position) |
| Selection Criteria | Gouty nephropathy | One claim for inpatient or outpatient ICD-9 diagnosis code 274.1x (any position) or ICD-10 diagnosis code M10.3x (any position) |
| Selection Criteria | Vasculitis | One claim for inpatient or outpatient ICD-9 diagnosis code 447.6 (any position) or ICD-10 diagnosis code I77.6 (any position) |
| Selection Criteria | Renal agenesis, dysgenesis, and hypoplasia | One claim for inpatient or outpatient ICD-9 diagnosis code 753.xx (any position) or ICD-10 diagnosis code Q60.x (any position) |
| Selection Criteria | Multiple myeloma | One claim for inpatient or outpatient ICD-9 diagnosis code 203.xx (any position) or ICD-10 diagnosis code C90.xx (any position) |
| Selection Criteria | Tubular necrosis | One claim for inpatient or outpatient ICD-9 diagnosis code 584.5 (any position) or ICD-10 diagnosis code N17.0x (any position) |
| Baseline Characteristics | Age (in categories) | 18-34, 35-44, 45-54,55-64, 65-74, 75-84, ≥85 and also assessed as 30, 30-39, 40-49, 50-59, 60-69, 70-79, ≥80 |
| Baseline Characteristics | Sex | Male, Female |
| Baseline Characteristics | Race | White, Asian, Black, Hispanic, Unknown |
| Baseline Characteristics | Region | Northeast: { CT, MA, ME, NH, NJ, NY, VT, RI, PA }  Midwest: { IL, IN, MI, IA, KS, MN, MO, NE, ND, SD, OH, WI } South: { DE, DC, FL, GA, MD, NC, AL, SC, VA, KY, MS, TN, WV, AR, LA, OK, TX }  West: { AZ, CO, ID, NM, MT, NV, AK, UT, WY, CA, HI, OR, WA } |
| Baseline Characteristics | Provider specialty | Endocrinologist, nephrologist, cardiologist, urologist, or (internal medicine specialist or family medicine/general, or internist/general internist or general practitioner or general practice specialist) |
| Comorbidity | Acute coronary syndrome or unstable angina | ICD-9 inpatient or outpatient diagnosis code 410.xx (any position) or ICD-10 inpatient or outpatient diagnosis code I21.xx or I22.xx (any position) (for myocardial infarction) or ICD-10 inpatient or outpatient diagnosis code I20.xx (any position) (for unstable angina) |
| Comorbidity | Acute kidney injury | ICD-9 inpatient diagnosis code 584.6x, 584.7x, 584.8x or 584.9x (any position) or ICD-10 inpatient diagnosis code N17.xx (excluding N17.0x) (any position) (Hwang 2012) |
| Comorbidity | Acute myocardial infarction | ICD-9 inpatient or outpatient diagnosis code 410.x (any position) or ICD-10 inpatient or outpatient diagnosis code I21.x, I22.x or I23.x (any position) |
| Comorbidity | Acute pancreatitis | ICD-9 inpatient or outpatient diagnosis code 577.0 (any position) or ICD-10 inpatient or outpatient diagnosis code K85.X (any position) (Reddy et al., 2016) |
| Comorbidity | Angina pectoris | ICD-9 inpatient or outpatient diagnosis code 413 or 414.0x (any position) or ICD-10 inpatient or outpatient diagnosis code I20.9 or I25.1x (any position) |
| Comorbidity | Anxiety disorder | ICD-9 inpatient or outpatient diagnosis code 293.84, 300.00, 300.01, 300.02, 300.09, 300.10, 300.20, 300.21, 300.22, 300.23, 300.29, 300.3, 300.5, 300.89, 300.9, 308.0, 308.1, 308.2, 308.3, 308.4, 308.9, 309.81, 313.0, 313.1, 313.21, 313.22, 313.3, 313.82 or 313.83 (any position) or ICD-10 inpatient or outpatient diagnosis code F06.4, F40.00, F40.01, F40.02, F40.10, F40.11, F40.210, F40.218, F40.220, F40.228, F40.230, F40.231, F40.232, F40.233, F40.240, F40.241, F40.242, F40.243, F40.248, F40.290, F40.291, F40.298, F40.8, F40.9, F41.0, F41.1, F41.3, F41.8, F41.9, F42, F43.0, F43.10, F43.11, F43.12, F48.8, F48.9, R45.2, R45.3, R45.4, R45.5, R45.6, R45.7, R45.81, R45.82, R45.83 or R45.84 (any position) (Elixhauser et al., 2018) |
| Comorbidity | Atherosclerosis | ICD-9 inpatient or outpatient diagnosis code 440.x (excluding 440.23 and 440.24) (any position) or ICD-10 inpatient or outpatient diagnosis code I70.x (excluding I70.25 and I70.26x) (any position) |
| Comorbidity | Atrial fibrillation | ICD-9 inpatient diagnosis code 427.31 (primary or secondary position) or ICD-10 inpatient diagnosis code I48.0, I48.1, I48.2 or I48.91 (primary or secondary position) (Chronic Conditions Data Warehouse, 2017) |
| Comorbidity | Coronary artery bypass grafting or percutaneous transluminal coronary angioplasty | ICD-9 inpatient procedure code 36.1, 36.10, 36.11, 36.12, 36.13, 36.14, 36.15, 36.16, 36.17 or 36.19 (any position) or CPT/HCPCS procedure code 33510, 33511, 33512, 33513, 33514, 33516, 33517, 33518, 33519, 33521, 33522, 33523, 33533, 33534, 33535 or 33536 or ICD-9 inpatient diagnosis code 414.04 or 414.07 (any position) or ICD-10 acute inpatient procedure code 0210083, 0210088, 0210089, 021008C, 021008F, 021008W, 0210093, 0210098, 0210099, 021009C, 021009F, 021009W, 02100A3, 02100A8, 02100A9, 02100AC, 02100AF, 02100AW, 02100J3, 02100J8, 02100J9, 02100JC, 02100JF, 02100JW, 02100K3, 02100K8, 02100K9, 02100KC, 02100KF, 02100KW, 02100Z3, 02100Z8, 02100Z9, 02100ZC, 02100ZF, 0210483, 0210488, 0210489, 021048C, 021048F, 021048W, 0210493, 0210498, 0210499, 021049C, 021049F, 021049W, 02104A3, 02104A8, 02104A9, 02104AC, 02104AF, 02104AW, 02104J3, 02104J8, 02104J9, 02104JC, 02104JF, 02104JW, 02104K3, 02104K8, 02104K9, 02104KC, 02104KF, 02104KW, 02104Z3, 02104Z8, 02104Z9, 02104ZC, 02104ZF, 0211088, 0211089, 021108C, 021108W, 0211098, 0211099, 021109C, 021109W, 02110A8, 02110A9, 02110AC, 02110AW, 02110J8, 02110J9, 02110JC, 02110JW, 02110K8, 02110K9, 02110KC, 02110KW, 02110Z8, 02110Z9, 02110ZC, 0211488, 0211489, 021148C, 021148W, 0211498, 0211499, 021149C, 021149W, 02114A8, 02114A9, 02114AC, 02114AW, 02114J8, 02114J9, 02114JC, 02114JW, 02114K8, 02114K9, 02114KC, 02114KW, 02114Z8, 02114Z9, 02114ZC, 021208C, 021208W, 021209C, 021209W, 02120AC, 02120AW, 02120JC, 02120JW, 02120KC, 02120KW, 02120ZC, 021248C, 021248W, 021249C, 021249W, 02124AC, 02124AW, 02124JC, 02124JW, 02124KC, 02124KW, 02124ZC, 021308C, 021308W, 021309C, 021309W, 02130AC, 02130AW, 02130JC, 02130JW, 02130KC, 02130KW, 02130ZC, 021348C, 021348W, 021349C, 021349W, 02134AC, 02134AW, 02134JC, 02134JW, 02134KC, 02134KW or 02134ZC (any position) or ICD-10 acute inpatient diagnosis code I25.810or I25.812 (any position) (for CABG) or ICD-9 inpatient procedure code 00.66 or 17.55 (any position) or CPT/HCPCS procedure code 92920, 92921, 92924, 92925, 92928, 92929, 92933 or 92934 or ICD-9 outpatient procedure code 00.66 or 17.55 (any position) or ICD-10 inpatient procedure code 02703ZZ, 02704ZZ, 02713ZZ, 02714ZZ, 02723ZZ, 02724ZZ, 02733ZZ, 02734ZZ or 02C03ZZ (any position) or ICD-10 outpatient procedure code 02703ZZ, 02704ZZ, 02713ZZ, 02714ZZ, 02723ZZ, 02724ZZ, 02733ZZ, 02734ZZ or 02C03ZZ (any position) (for PTCA) |
| Comorbidity | Coronary artery disease | ICD-9 inpatient or outpatient diagnosis code 410.x 411.1, 412 or 413.9 (any position) or ICD-10 inpatient or outpatient diagnosis code I20.0, I21.x, I22.x, I24.x, I25.1x-I25.2 or I25.8x-I25.9 (any position) |
| Comorbidity | Chronic lung/pulmonary disease | ICD-9 inpatient or outpatient diagnosis code 416.8, 416.9, 490.x–505.x, 506.4, 508.1 or 508.8 (any position) or ICD-10 inpatient or outpatient diagnosis code I27.8, I27.9, J40.x–J47.x, J60.x–J67.x, J68.4, J70.1 or J70.3 (any position) (Quan et al, 2005) |
| Comorbidity | Depression | ICD-9 inpatient or outpatient diagnosis code 296.2, 296.3, 298.0, 300.4, 309.0, 309.1, 309.28 or 311 (any position) or ICD-10 inpatient or outpatient diagnosis code F20.4, F31.3–F31.5, F32.x, F33.x, F34.1, F43.21 or F43.23 (any position) (Quan et al, 2005; Townsend et al, 2012) |
| Comorbidity | Diabetic retinopathy | ICD-9 inpatient or outpatient diagnosis code 362.xx (any position) or ICD-10 diagnosis code E08.3x, E11.3x or E13.3x (any position) |
| Comorbidity | Edema | ICD-9 inpatient or outpatient diagnosis code 782.3 (any position) or ICD-10 inpatient or outpatient diagnosis code R60.x (any position) (Chinali et al, 2010) |
| Comorbidity | Fatigue & sleep related disorders | ICD-9 inpatient or outpatient diagnosis code 780.7x, 300.5x, 780.50, 780.52, 780.54-780.56, 780.58-.59, 307.4x, 327.0x or 327.1x (any position) or ICD 10 inpatient or outpatient diagnosis code R53.1, R53.8x, F48.8, F51.02, F51.09, F51.01, F51.03, F51.04, F51.05, F51.19, F51.11, F51.12, F51.13, F51.8, G47.0x, G47.1x, G47.2x, G47.4x, G47.5x, G47.6x, G47.8, G47.9 or G93.3 (any position) |
| Comorbidity | Heart failure | ICD-9 inpatient or outpatient diagnosis code 398.91, 402, 402.01, 402.11, 402.91, 404.01, 404.03, 404.11, 404.13, 404.91, 404.93 or 428.xx (primary or secondary position) or ICD-10 inpatient or outpatient diagnosis code I09.81, I11.0, I13.0, I13.2, I50.1. I50.20, I50.21, I50.22, I50.23, I50.30, I50.31, I50.32, I50.33, I50.40, I50.41, I50.42, I50.43 or I50.9 (primary or secondary position) |
| Comorbidity | Hemorrhagic stroke | ICD-9 inpatient diagnosis code 430, 431, 432, 432.0, 432.1 or 432.9 (any position) or ICD-10 inpatient diagnosis code I60, I60.0, I60.00, I60.01, I60.02, I60.1, I60.10, I60.11, I60.12, I60.2, I60.20, I60.21, I60.22, I60.3, I60.30, I60.31, I60.32, I60.4, I60.5, I60.50, I60.51, I60.52, I60.6, I60.7, I60.8, I60.9, I61, I61.0, I61.1, I61.2, I61.3, I61.4, I61.5, I61.6, I61.8, I61.9, I62, I62.0, I62.00, I62.01, I62.02, I62.03, I62.1 or I62.9 (any position) |
| Comorbidity | History of myocardial infarction | ICD-9 inpatient or outpatient diagnosis code 410.xx (any position) or ICD-10 inpatient or outpatient diagnosis code I21.xx or I22.xx (any position) (Sun et al, 2017; American Diabetes Association, 2018) |
| Comorbidity | Hyperkalemia | ICD-9 inpatient or outpatient diagnosis code 276.7 (any position) or ICD-10 inpatient or outpatient diagnosis code E87.5 (any position) or LOINC code is any of {12812-4, 12813-2, 22760-3, 2823-3, 2824-1, 29349- 8, 42569-4, 51618-7, 6298-4, 75940-7, 77142-8 } and value is >5.5 |
| Comorbidity | Hyperlipidemia | ICD-9 inpatient or outpatient diagnosis code 272.0-272.4 (any position) or ICD-10 inpatient or outpatient diagnosis code E78.0-E78.5 (any position) (Bellows et al, 2017) |
| Comorbidity | Hypertension | ICD-9 inpatient or outpatient diagnosis code 401.x-405.x (any position) or ICD-10 inpatient or outpatient diagnosis code I10.x-I13.x or I15.x (any position) (Tonelli et al, 2015) |
| Comorbidity | Hypoglycemia | ICD-9 inpatient or outpatient diagnosis code 251.0, 251.1, 251.2, 270.3, 775.0, 775.6, 962.3, 250.3 or 250.8 (any position) or ICD-10 inpatient or outpatient diagnosis code E15, E16.0, E16.1, E16.2, E71.0, E71.120, E71.19, E71.2, P70.0, P70.1, P70.4, T38.3X1A, T38.3X2A, T38.3X3A or T38.3X4A (any position) |
| Comorbidity | Hypokalemia | ICD-9 inpatient or outpatient diagnosis code 276.8 (any position) or ICD-10 inpatient or outpatient diagnosis code E87.6 (any position) (Ginde et al., 2008) |
| Comorbidity | Hyponatremia | ICD-9 inpatient or outpatient diagnosis code 276.1 (any position) (Shea et al., 2008; Movig et al., 2003) or ICD-10 inpatient or outpatient diagnosis code E87.1 (any position) (Gandhi et al., 2017) |
| Comorbidity | Ischemic stroke | ICD-9 inpatient diagnosis code 433.01, 433.11, 433.21, 433.31, 433.81, 433.91, 434.01, 434.11 or 434.91 (any position) or ICD-10 inpatient diagnosis code I63, I63.0, I63.00, I63.01, I63.011, I63.012, I63.019, I63.02, I63.03, I63.031, I63.032, I63.039, I63.09, I63.1, I63.10, I63.11, I63.111, I63.112, I63.119, I63.12, I63.13, I63.131, I63.132, I63.139, I63.19, I63.2, I63.20, I63.21, I63.211, I63.212, I63.219, I63.22, I63.23, I63.231, I63.232, I63.239, I63.29, I63.3, I63.30, I63.31, I63.311, I63.312, I63.319, I63.32, I63.321, I63.322, I63.329, I63.33, I63.331, I63.332, I63.339, I63.34, I63.341, I63.342, I63.349, I63.39, I63.4, I63.40, I63.41, I63.411, I63.412, I63.419, I63.42, I63.421, I63.422, I63.429, I63.43, I63.431, I63.432, I63.439, I63.44, I63.441, I63.442, I63.449, I63.49, I63.5, I63.50, I63.51, I63.511, I63.512, I63.519, I63.52, I63.521, I63.522, I63.529, I63.53, I63.531, I63.532, I63.539, I63.54, I63.541, I63.542, I63.549, I63.59, I63.6, I63.8 or I63.9 (any position) |
| Comorbidity | Late effects cerebrovascular disease | ICD-9 inpatient or outpatient diagnosis code 438.xx (any position) or ICD-10 inpatient or outpatient diagnosis code I69.xx (any position) |
| Comorbidity | Lower extremity amputation | ICD-9 acute inpatient procedure code 84.1x (any position) or ICD-9 outpatient procedure code 84.1x (primary position) or ICD-10 acute inpatient procedure code 0Y620ZZ, 0Y630ZZ, 0Y640ZZ, 0Y670ZZ, 0Y680ZZ, 0Y6C0Z1, 0Y6C0Z2, 0Y6C0Z3, 0Y6D0Z1, 0Y6D0Z2, 0Y6D0Z3, 0Y6F0ZZ, 0Y6G0ZZ, 0Y6H0Z1, 0Y6H0Z2, 0Y6H0Z3, 0Y6J0Z1, 0Y6J0Z2, 0Y6J0Z3, 0Y6M0Z0, 0Y6M0Z4, 0Y6M0Z5, 0Y6M0Z6, 0Y6M0Z7, 0Y6M0Z8, 0Y6M0Z9, 0Y6M0ZB, 0Y6M0ZC, 0Y6M0ZD, 0Y6M0ZF, 0Y6N0Z0, 0Y6N0Z4, 0Y6N0Z5, 0Y6N0Z6, 0Y6N0Z7, 0Y6N0Z8, 0Y6N0Z9, 0Y6N0ZB, 0Y6N0ZC, 0Y6N0ZD, 0Y6N0ZF, 0Y6P0Z0, 0Y6P0Z1, 0Y6P0Z2, 0Y6P0Z3, 0Y6Q0Z0, 0Y6Q0Z1, 0Y6Q0Z2, 0Y6Q0Z3, 0Y6R0Z0, 0Y6R0Z1, 0Y6R0Z2, 0Y6R0Z3, 0Y6S0Z0, 0Y6S0Z1, 0Y6S0Z2, 0Y6S0Z3, 0Y6T0Z0, 0Y6T0Z1, 0Y6T0Z2, 0Y6T0Z3, 0Y6U0Z0, 0Y6U0Z1, 0Y6U0Z2, 0Y6U0Z3, 0Y6V0Z0, 0Y6V0Z1, 0Y6V0Z2, 0Y6V0Z3, 0Y6W0Z0, 0Y6W0Z1, 0Y6W0Z2, 0Y6W0Z3, 0Y6X0Z0, 0Y6X0Z1, 0Y6X0Z2, 0Y6X0Z3, 0Y6Y0Z0, 0Y6Y0Z1, 0Y6Y0Z2, 0Y6Y0Z3 (any position) |
| Comorbidity | Microvascular complications disease | ICD 9 inpatient or outpatient diagnosis code 250.6x or 357.2 (any position) or ICD-10 inpatient or outpatient diagnosis code E11.4x (Boulanger et al, 2009) (for neuropathy) or ICD-9 inpatient or outpatient diagnosis code 250.40, 250.42, or 583.xx (any position) or ICD-10 inpatient or outpatient diagnosis code E11.21, E13.21, E08.21, N07.xx, or N08 (any position) (Fleet et al, 2013) (for nephropathy) or ICD-9 inpatient or outpatient diagnosis code 362.xx (any position) or ICD-10 inpatient or outpatient diagnosis code E08.3x, E11.3x or E13.3x (any position) (for retinopathy) |
| Comorbidity | Obesity or weight gain (as identified via ICD codes and procedure codes) | ICD-9 inpatient or outpatient diagnosis code 278.xx (any position), ICD-10 inpatient or outpatient diagnosis code E66.xx, Z68.3x or Z68.4x (any position), or HCPCS procedure code G0447 or G0473 (ACOG Obesity Toolkit, 2016) |
| Comorbidity | Obstructive sleep apnea | ICD-9 inpatient or outpatient diagnosis code 327.23 (any position) or ICD-10 inpatient or outpatient diagnosis code G47.33 (any position) |
| Comorbidity | Osteoarthritis | ICD-9 inpatient or outpatient diagnosis code 715.xx or V13.4 (any position) or ICD-10 inpatient or outpatient diagnosis code M15.0, M15.1, M15.2, M15.3, M15.4, M15.8, M15.9, M16.0, M16.10, M16.11, M16.12, M16.2, M16.30, M16.31, M16.32, M16.4, M16.50, M16.51, M16.52, M16.6, M16.7, M16.9, M17.0, M17.10, M17.11, M17.12, M17.2, M17.30, M17.31, M17.32, M17.4, M17.5, M17.9, M18.0, M18.10, M18.11, M18.12, M18.2, M18.30, M18.31, M18.32, M18.4, M18.50, M18.51, M18.52, M18.9, M19.011, M19.012, M19.019, M19.021, M19.022, M19.029, M19.031, M19.032, M19.039, M19.041, M19.042, M19.049, M19.071, M19.072, M19.079, M19.111, M19.112, M19.119, M19.121, M19.122, M19.129, M19.131, M19.132, M19.139, M19.141, M19.142, M19.149, M19.171, M19.172, M19.179, M19.211, M19.212, M19.219, M19.221, M19.222, M19.229, M19.231, M19.232, M19.239, M19.241, M19.242, M19.249, M19.271, M19.272, M19.279, M19.90, M19.91, M19.92 or M19.93 (any position) |
| Comorbidity | Pain disorders | ICD-9 inpatient or outpatient diagnosis code 720.xx, 721.2x to 721.9x, 722.1x to 722.3x, 722.5x, 722.6x, 722.70, 722.72, 722.73, 722.80, 722.82, 722.83, 722.90, 722.92, 722.93, 724.xx, 729.1x, 780.96, 338.xx, 307.80, 053.1x, 250.6x, 307.8x, 323.xx, 335.20, 335.34, 336.9x, 337.1x, 337.2x, 338.3x, 339.xx, 340.xx, 341.xx, 346.0x to 346.9x, 350.xx, 351.xx, 353.xx to 356.xx, 357.2x, 358.xx, 524.6x, 577.1x, 696.xx, 714.xx, 715.xx, 719.xx, 720.xx, 721.0x to 722.4x, 722.71, 722.81, 722.91, 723.1x, 724.4x, 728.0x, 729.0x, 729.2x, 729.5x, 784.0x, 786.5x, 789.xx, 733.99, 733.14, 780.71, 820.8x, 820.9x, 951.4x, 952.xx, 953.4x or 955.5x-955.7x (any position) or ICD-10 inpatient or outpatient diagnosis code E08.42, E09.42, E11.42, E13.42, G04.xx, G05.xx, G35, G36.xx, G37.xx, G43.xx, G44.xx, G50.xx, G51.xx, G54.xx, G56.xx, G57.xx, G58.7, G60.xx, G89.xx, G90.xx, G95.xx, G99.xx, M05.xx, M06.xx, M12.xx, M26.6xx, M43.xx, M45.xx, M46.xx, M47.xx, M48.xx, M50.xx, M51.xx, M53.xx, M54.xx, M60.xx, M79.xx, M96.1, R07.xx, R10.xx, R16.xx, R19.xx, R51, R52, F45.4x, B02.2x, S04.1x, S14.1x, S24.1x, S34.1x, S34.3x, S44.xx, S54.xx, S64.xx or S74.xx (any position) |
| Comorbidity | Pneumonia | ICD-9 inpatient or outpatient diagnosis code 481.xx-484.xx (any position) or ICD-10 inpatient or outpatient diagnosis code 22.1, A37.91, A48.1, B25.0, B44.0, J13, J14, J15.0, J15.1, J15.20, J15.211, J15.212, J15.29, J15.3, J15.4, J15.5, J15.6, J15.7, J15.8, J15.9, J16.0, J16.8, J17, J18.0, J18.1 or J18.9 (any position) |
| Comorbidity | Prevalent anemia | ICD-9 inpatient or outpatient diagnosis code 280.xx-285.xx (any position) or ICD-10 inpatient or outpatient diagnosis code D50.0, D50.1, D50.8, D50.9, D51.0, D51.1, D51.3, D51.8, D52.0, D52.1, D52.8, D52.9, D53.0, D53.1, D53.2, D53.8, D53.9, D55.0, D55.1, D55.8, D56.0, D56.1, D56.2, D56.3, D56.4, D56.5, D56.8, D56.9, D57.00, D57.1, D57.20, D57.219, D57.3, D57.40, D57.419, D57.80, D57.819, D58.0, D58.1, D58.2, D58.8, D58.9, D59.0, D59.1, D59.3, D59.4, D59.5, D59.6, D59.8, D59.9, D60.0, D60.1, D60.8, D60.9, D61.01, D61.09, D61.1, D61.2, D61.810, D61.811, D61.818, D61.82, D61.89, D61.9, D62, D63.0, D63.1, D63.8, D64.0, D64.1, D64.2, D64.3, D64.4, D64.81, D64.89 or D64.9 (any position) |
| Comorbidity | Prior cardiac procedure | CPT/HCPCS code 92920-93799 or other codes for cardiac procedure variables in this study |
| Comorbidity | Proteinuria | ICD-9 inpatient or outpatient diagnosis code 791.0 (any position) or ICD-10 inpatient or outpatient diagnosis code R80.3 or R80.9 (any position) |
| Comorbidity | Peripheral vascular disease | ICD-9 inpatient or outpatient diagnosis code 093.0, 437.3, 440.x, 441.x, 443.x, 447.1, 557.1, 557.9 or V43.4 (any position) or ICD-10 inpatient or outpatient diagnosis code 170.x, I71.x, I73.xx, I77.1, I79.0, I79.2, K55.1, K55.8, K55.9, Z95.8 or Z95.9 (any position) (Quan et al, 2005) |
| Comorbidity | Respiratory failure | ICD-9 inpatient diagnosis code 518.5x, 518.81, 518.83 or 518.84 (any position) or ICD-10 inpatient diagnosis code J96.xx (any position) (Behrendt, 2000) |
| Comorbidity | Resistant hypertension | Pharmacy claims for three or more unique antihypertensive medications or combination medications with the following NDC generic names: benazepril hydrochloride, captopril, enalapril, maleate, fosinopril sodium, lisinopril, moexipril, perindopril, quinapril hydrochloride, ramipril, trandolapril, candesartan, eprosarta, mesylate, irbesarten, losartan potassium, telmisartan, valsartan, acebutolol, atenolol, betaxolol, bisoprolol fumarate, carteolo, hydrochloride, metoprolol tartrate, metoprolol, succinate, nadolol, penbutolol sulfate, pindolol, propranolol hydrochloride, solotol hydrochloride, timolol maleate, chlorthalidone, chlorothiazide, hydrochlorothiazide, indapamide, metolazone, amiloride hydrochloride, triamterene, furosemide, bumetanide, amlodipine besylate, bepridil, diltiazem hydrochloride, felodipine, isradipine, nicardipine, nifedipine, nisoldipine, verapamil hydrochloride, Spironolactone, Eplerenone, doxazosin mesylate, prazosin hydrochloride, terazosin hydrochloride, methyldopa, alpha methyldopa, clonidine hydrochloride, guanabenz acetate, guanfacine hydrochloride, guanadrel, guanethidine, monosulfate, Reserpine, hydralazine hydrochloride, minoxidil (American College of Cardiology, 2017; Braam et al, 2016) |
| Comorbidity | Sleep apnea | ICD-9 inpatient or outpatient diagnosis code 327.2x, 780.51, 780.53 or 780.57 (any position) or ICD-10 inpatient or outpatient diagnosis code G47.3x (any position) (AFHSC Surveillance, 2017) |
| Comorbidity | Stroke | ICD-9 inpatient diagnosis code 430.x-431.x, 433.x1, 434.x1 or 436.x (any position) or ICD-10 inpatient diagnosis code I60.x, I61.x-I63.x or I67.89 (any position) (Andrade et al, 2012; Sacco et al, 2013) |
| Comorbidity | Transient ischemic attack | ICD-9 inpatient or outpatient diagnosis code 435.x (any position) or ICD-10 inpatient or outpatient diagnosis code G45.x or I67.848 (any position) |
| Comorbidity | Lifestyle factor | Definition |
| Comorbidity | Smoking and  nicotine  dependence | CPT/HCPCS code 0002F, 0004F, 1034F, 4000F, 4001F, C9801, D1320, G0375, G0376, G0436, G0437, G8455, 1032F, C9802, G9276 or ICD-10 inpatient or outpatient code F17.20, F17.200, F17.201, F17.203, F17.208, F17.209, F17.21, F17.210, F17.211, F17.213, F17.218, F17.219, F17.29, F17.290, F17.291, F17.293, F17.298, F17.299, Z72.0, 292.0 (any position) |
| Comorbidity | Smoking cessation | NDC WHO ATC code N06AX12, N07BA01 or N07BA03 |
| Comorbidity | Alcohol abuse | ICD-9 inpatient or outpatient diagnosis code 291.1, 291.2, 291.5, 291.8, 291.81, 291.82, 291.89, 291.9, 303.90, 303.91, 303.92, 303.93, 305.00, 305.01, 305.02, 305.03 or V11.3 (any position) or ICD-10 inpatient or outpatient diagnosis code F10.x, E52.x, G62.1, I42.6, K29.2, K70.0, K70.3, K70.9, T51.x, Z50.2, Z71.4 or Z72.1 (any position) |
| Comorbidity | Influenza vaccination | CPT/HCPCS code 90630, 90674, 90682, 90685-90688, 90756, 90656, 90658, 90673, 90460, 90471-90474 |
| Laboratory  values | eGFR | LOINC code is any of { 9318-7, 14959-1, 30000-4, 14958-3, 32294-1 } |
| Laboratory  values | UACR | LOINC code is any of { 33914-3, 48642-3, 48643-1, 50044-7, 50210-4, 50384-7, 62238-1, 69405-9, 70969-1, 76633-7, 77147-7 } |
| Laboratory  values | HbA1c | LOINC code is any of { 17856-6, 4548-4, 4549-2, 59261-8, 62388-4, 71875- 9, 74246-0 } |
| Medication (based on NDC generic names) | Angiotensin converting enzyme inhibitor | Benazepril hydrochloride, captopril, enalapril maleate, fosinopril sodium, lisinopril, moexipril, perindopril, quinapril hydrochloride, ramipril, trandolapril |
| Medication (based on NDC generic names) | Anticoagulant | Apixaban, ardeparin sodium,porcine, argatroban, argatroban in 0.9 % sodium chloride, argatroban in sodium chloride, iso-osmotic  bivalirudin, dabigatran etexilate mesylate, dalteparin sodium,porcine danaparoid sodium,porcine, desirudin, edoxaban tosylate, enoxaparin sodium, fondaparinux sodium, heparin sodium,beef, heparin sodium,porcine, heparin sodium,porcine in 0.45 % sodium chloride, heparin sodium,porcine in 0.9 % sodium chloride, heparin sodium,porcine in 0.9 % sodium chloride/pf, heparin sodium,porcine/dextrose 5 % in water, heparin sodium,porcine/dextrose 5 % in water/pf, heparin sodium,porcine/pf, lepirudin,recombinant, rivaroxaban, tinzaparin sodium,porcine, warfarin sodium |
| Medication (based on NDC generic names) | Antiplatelet agent | Aspirin, aspirin (calcium carb & magnesium buffers)/pravastatin, aspirin/acetaminophenaspirin/acetaminophen/caffeine, aspirin/acetaminophen/caffeine/calcium, aspirin/acetaminophen/caffeine/ potassium, aspirin/acetaminophen/calcium carbonate, aspirin/ acetaminophen/magnesium/aluminum hydroxide/caffeine, aspirin/ caffeine, aspirin/calcium carbonate, aspirin/calcium carbonate/ magnesium, aspirin/calcium carbonate/magnesium/aluminum hydroxide, aspirin/codeine phosphate, aspirin/diphenhydramine citrate, aspirin/ diphenhydramine HCL, aspirin/diphenhydramine/sodium bicarbonate/ citric acid, aspirin/dipyridamole, aspirin/magnesium carbonate/ dihydroxyaluminum aminoacetate, aspirin/magnesium hydroxide/ aluminum hydroxide, aspirin/magnesium hydroxide/ aluminum hydroxide/caffeine, aspirin/meprobamate, aspirin/ salicylamide/ acetaminophen/caffeine, aspirin/salicylamide/ caffeine, aspirin/sodium bicarbonate/citric acid, cilostazol, clopidogrel bisulfate, dipyridamole, prasugrel HCL, ticagrelor,vorapaxar sulfate, abciximab, butalbital/ aspirin/caffeine, carisoprodol/aspirin, chlorpheniramine mal/phenyl-ephrine/d-methorphan hb/aspirin, chlorpheniramine maleate/ phenyl-ephrine bitartrate/aspirin, cinnamedrine HCL/aspirin/ caffeine, codeine phosphate/butalbital/aspirin/caffeine, codeine phosphate/ carisoprodol/ aspirin, codeine/aspirin/salicylamide/acetaminophen/ caffeine, dihydrocodeine bitartrate/aspirin/caffeine, dihydrocodeine/ aspirin/ caffeine, ephedrine/aspirin/acetanilide/caffeine, hydrocodone bitartrate/ aspirin, methocarbamol/aspirin, orphenadrine citrate/aspirin/ caffeine, oxycodone HCL/aspirin, oxycodone HCL/oxycodone terephthalate/ aspirin, oxycodone/aspirin, pentazocine HCL/aspirin, phenylephrine HCL/aspirin, phenylpropanolamine bitartrate/aspirin, phenyl-propano-lamine bitartrate/aspirin/ chlorpheniramine, phenylpropanolamine HCL/aspirin, phenylpropanolamine HCL/aspirin/ chlorpheniramine, phenylpropanolamine HCL/aspirin/chlorpheniramine/ caffeine, phenylpropanolamine HCL/aspirin/diphenhydramine, propoxy-phene HCL/aspirin/caffeine, pseudoephedrine HCL/aspirin/ chlorpheniramine |
| Medication (based on NDC generic names) | Alpha blocker | Doxazosin mesylate, prazosin hydrochloride, terazosin hydrochloride |
| Medication (based on NDC generic names) | Alpha glucosidase inhibitor | Acarbose, miglitol |
| Medication (based on NDC generic names) | Angiotensin II receptor blocker | Candesartan, eprosartan mesylate, irbesarten, losartan potassium, telmisartan, valsartan |
| Medication (based on NDC generic names) | Aspirin | Aspirin |
| Medication (based on NDC generic names) | Basal insulin  (includes long  acting and  intermediate  acting insulins) | Insulin detemir, insulin glargine, human recombinant analog, insulin degludec, insulin isophane nph,bf-pk, insulin isophane, beef, insulin isophane, beef pure, insulin isophane, pork pure, insulin nph human semi syn, insulin nph human semi-syn/insulin reg human semi-syn, insulin zinc beef, insulin zinc extend human rec, insulin zinc extended, bf-pk, insulin zinc human rec, insulin zinc human semi-syn, insulin zinc prompt, bf-pk, insulin zinc, beef purified, insulin zinc, beef purified/insulin zinc, pork purified, insulin zinc, beef-pork, insulin zinc, pork purified, insulin, pork purified/insulin isophane, pork pure, nph, human insulin isophane, insulin, beef, insulin, pork, insulin, pork purified |
| Medication (based on NDC generic names) | Beta blocker | Acebutolol, atenolol, betaxolol, bisoprolol fumarate, carteolol, hydrochloride, metoprolol tartrate, metoprolol succinate, nadolol, penbutolol sulfate, pindolol, propranolol hydrochloride, solotol hydrochloride, timolol maleate, carvedilol, nebivolol, labetalol, esmolol, carvedilol, nebivolol, labetalol, esmolol |
| Medication (based on NDC generic names) | Calcium channel blocker | Amlodipine besylate, bepridil, diltiazem, hydrochloride, felodipine, isradipine, nicardipine, nifedipine, nisoldipine, verapamil hydrochloride |
| Medication (based on NDC generic names) | Centrally acting alpha agonist | Guanadrel, methyldopa, clonidine hydrochloride, guanabenz acetate, guanfacine hydrochloride |
| Medication (based on NDC generic names) | Digoxin | Digoxin |
| Medication (based on NDC generic names) | Direct renin inhibitor | Aliskiren |
| Medication (based on NDC generic names) | DPP4i | Sitagliptin, saxagliptin, linagliptin, alogliptin |
| Medication (based on NDC generic names) | Epithelial sodium channel blockers | Amiloride hydrochloride, triamterene |
| Medication (based on NDC generic names) | Erythropoietin stimulating agent | Darbepoetin alfa, epoetin alfa, methoxy polyethylene glycol-epoetin beta, erythropoietin |
| Medication (based on NDC generic names) | GLP1ra | Exenatide, liraglutide, albiglutide, dulaglutide, semaglutide, lixisenatide |
| Medication (based on NDC generic names) | HMG-CoA reductase inhibitor (statin) | Atorvastatin, fluvastatin, lovastatin, pitavastatin, pravastatin, rosuvastatin, simvastatin |
| Medication (based on NDC generic names) | Loop acting diuretic | Furosemide, bumetanide, torsemide, ethacrynic acid |
| Medication (based on NDC generic names) | Mealtime insulin (includes short acting and rapid acting insulins) | Insulin lispro, insulin lispro protamine & insulin lispro, insulin aspart, insulin aspart protamine human/insulin aspart, insulin glulisine, insulin protamine zinc, beef, insulin protamine zinc, beef (p), insulin protamine zinc, bf-pk, insulin reg human semi-syn, insulin reg, hum s-s buff, insulin regular, human, insulin regular, human/insulin release unit, insulin regular, human/insulin release unit/chamber/inhaler, insulin regular, beef-pork, insulin regular, human buffered, insulin, pork reg. concentrate, nph, human insulin isophane/insulin regular, human |
| Medication (based on NDC generic names) | Meglinitide | Repaglinide, nateglinide |
| Medication (based on NDC generic names) | Metformin | Metformin |
| Medication (based on NDC generic names) | Mineralocorticoid receptor antagonist | Spironolactone, eplerenone |
| Medication (based on NDC generic names) | Nitrate | Nitroglycerin sublingual tablet, isosorbide mononitrate and dinitrate, nitroglycerin intravenous, nitroglycerin lingual, nitroglycerin lingual aerosol, nitroglycerin oral capsule, nitroglycerin sublingual tablet, nitroglycerin transdermal infusion system, nitroglycerin transdermal ointment, nitroglycerin transdermal therapeutic system, nitrolinglycerin pumpspray |
| Medication (based on NDC generic names) | Oral anticoagulant | Warfarin sodium, dabigatran etexilate, rivaroxaban, apixaban, edoxaban tosylate |
| Medication (based on NDC generic names) | Potassium binding agent | Sodium polystyrene sulfonate, patiromer |
| Medication (based on NDC generic names) | Potassium-sparing diuretic | Eplerenone, triamterene, spironolactone, amiloride |
| Medication (based on NDC generic names) | Potassium supplement | Potassium chloride |
| Medication (based on NDC generic names) | Sulfonylurea | Chlorpropamide, glipizide, glyburide, and glimepiride |
| Medication (based on NDC generic names) | Thiazide diuretic | Chlorthalidone, chlorothiazide, hydrochlorothiazide, indapamide, metolazone |
| Medication (based on NDC generic names) | Thiazolidinedione | Rosiglitazone, pioglitazone |
| Healthcare Resource Utilization Definitions | Number of hospitalizations | Count of any events within the inpatient confinement |
| Healthcare Resource Utilization Definitions | Duration of inpatient stay | Sum of duration of all events related to ICD-9 or ICD-10 inpatient diagnosis code within the inpatient confinement (any position) |
| Healthcare Resource Utilization Definitions | Number of outpatient encounters | Count of events with any ICD-9 or ICD-10 outpatient diagnosis code (any position) and type of service “office visit” or “outpatient” (any position) occurring on different days |
| Healthcare Resource Utilization Definitions | Number of ED encounters | Count of events with any ICD-9 or ICD-10 diagnosis code (any position) and type of service code FAC_OP.ER or PROF.ER (any position) or revenue code 0450, 0451, 0452, 0456, 0459 or 0981 |
| Healthcare Resource Utilization Definitions | Number of different medications used (by generic name) | Count of number of different NDC generic names across the baseline period |
| Healthcare Resource Utilization Definitions | Number of different medication classes for T2D | Count of number of different T2D classes (identified by NDC generic names) across the baseline period |
| Clinical Outcomes | CV hospitalization as defined by MI, stroke, and HF diagnosis codes | ICD-9 inpatient diagnosis code 410.xx or ICD-10 inpatient or outpatient diagnosis code I21.xx or I22.xx (primary or secondary position) (Sun et al, 2017; American Diabetes Association 2018) (for MI) or ICD-9 inpatient diagnosis code 430.x-431.x, 433.x1, 434.x1 or 436.x or ICD-10 inpatient diagnosis code I60.x, I61.x-I63.x or I67.89 (primary or secondary position) (Andrade et al, 2012; Sacco et al, 2013) (for stroke) or ICD-9 inpatient diagnosis code 398.91, 402.01, 402.11, 402.91, 404.01, 404.03, 404.11, 404.13, 404.91, 404.93 or 428.xx or ICD-10 inpatient diagnosis code I09.81, I11.0, I13.0, I13.2, I50.1. I50.20, I50.21, I50.22, I50.23, I50.30, I50.31, I50.32, I50.33, I50.40, I50.41, I50.42, I50.43 or I50.9 (primary or secondary position) (for HF) |
| Clinical Outcomes | Renal hospitalization as defined by CKD diagnosis codes | ICD-9 inpatient diagnosis code 250.4, 285.21, 403, 403.00, 403.01, 403.10, 403.11, 403.90, 403.91, 404, 404.00, 404.01, 404.02, 404.03, 404.10, 404.11, 404.12, 404.13, 404.90, 404.91, 404.92, 404.93, 585, 585.1, 585.2, 585.3, 585.4, 585.5, or 585.9 (primary or secondary position) or ICD-10 inpatient diagnosis code D63.1, E082.2, E092.2, E102.2, E112.2, E132.2, I12, I12.0, I12.9, I13, I13.0, I13.1, I13.10, I13.11, I13.2, N18, N18.1, N18.2, N18.3, N18.4, N18.5, N18.6 or N18.9 (primary or secondary position) |
| Clinical Outcomes | Acute kidney injury hospitalization | ICD-9 inpatient diagnosis code 584.6x, 584.7x, 584.8x or 584.9x (any position) or ICD-10 inpatient diagnosis code N17.xx (excluding N17.0x) (any position) (Hwang 2012) |
| Clinical Outcomes | SGLT2i (for persistence and treatment failure outcome) | NDC generic name for canagliflozin, dapagliflozin propanediol, empagliflozin, canagliflozin/metformin HCL, dapagliflozin  propanediol/metformin HCL, empagliflozin/metformin HCL, dapagliflozin propanediol/saxagliptin HCL, or empagliflozin/linagliptin |

# **Additional File 1: Table 2: Overall Multivariate Time Dependent Assessment of Factors Associated with Treatment Failure among SGLT2i initiators with DKD and stratified by insurance**

| Variable | Hazard Ratio | p-value |
| --- | --- | --- |
| Overall Population |  |  |
| DPP4i | 1.33 (1.23, 1.43) | <0.01 |
| GLP1ra | 0.8 (0.72, 0.88) | <0.01 |
| Basal insulin | 0.79 (0.73, 0.86) | <0.01 |
| GLP1ra + basal insulin | 0.77 (0.64, 0.93) | 0.01 |
| Statin | 0.9 (0.82, 0.98) | 0.02 |
| Number of different medications by generic name | 0.99 (0.99, 1.0) | <0.01 |
| Diabetic ketoacidosis | 2.43 (1.17, 5.07) | 0.02 |
| Commercially Insured Patients |  |  |
| Acidosis | 3.02 (1.23, 7.44) | 0.02 |
| Diabetic ketoacidosis | 2.95 (0.9, 9.74) | 0.08 |
| AKI hospitalization | 0.0 (0.0, 0.0) | <0.01 |
| DPP4i | 1.6 (1.41, 1.82) | <0.01 |
| GLP1ra | 0.74 (0.63, 0.87) | 0 |
| GLP1ra + basal insulin | 0.62 (0.47, 0.81) | 0 |
| Statin | 0.83 (0.72, 0.96) | 0.01 |
| Duration of inpatient stay | 0.98 (0.95, 1.0) | 0.03 |
| Number of different medications by generic | 0.99 (0.98, 1.0) | 0.01 |
| Medicare Patients |  |  |
| Lower extremity amputation | 0.0 (0.0, 0.0) | <0.01 |
| Volume depletion | 1.61 (1.06, 2.44) | 0.03 |
| General practitioner/Internal medicine | 1.15 (1.05, 1.27) | <0.01 |
| Urologist | 1.91 (1.09, 3.33) | 0.02 |
| Outpatient facility | 1.46 (1.07, 2.01) | 0.02 |
| DPP4i | 1.19 (1.09, 1.3) | <0.01 |
| GLP1ra | 0.81 (0.72, 0.92) | <0.01 |
| Basal insulin | 0.71 (0.64, 0.78) | <0.01 |

# **Additional File 1: Table 3: Adverse Events of Interest over Follow-up**

| SGLT2i initiators with DKD (N = 6,389) | Patients With Events | | Number of Events | |
| --- | --- | --- | --- | --- |
|  | No Treatment Failure  n=1,587 | Treatment Failure n=4,802 | No Treatment Failure | Treatment Failure |
| Acidosis | 2 (0.1%) | 145 (3.0%) | 17 | 297 |
| AKI hospitalization | 0 (0.0%) | 133 (2.8%) | 9 | 218 |
| Dehydration | 3 (0.2%) | 221 (4.6%) | 26 | 452 |
| Diabetic ketoacidosis | 1 (0.1%) | 43 (0.9%) | 8 | 80 |
| Genital tract infection | 12 (0.8%) | 748 (15.6%) | 86 | 1857 |
| Hypotension | 1 (0.1%) | 172 (3.6%) | 16 | 377 |
| Lower extremity amputation | 1 (0.1%) | 16 (0.3%) | 1 | 27 |
| Urinary tract infection | 9 (0.6%) | 648 (13.5%) | 74 | 1634 |
| Volume depletion | 3 (0.2%) | 249 (5.2%) | 30 | 502 |
