# Supplementary material for: Predictors of cardio-kidney complications and treatment failure in patients with chronic kidney disease and type 2 diabetes treated with SGLT2 inhibitors
Source: BMC Med. 2022 Jan 10;20:2. doi: 10.1186/s12916-021-02191-2 (PMC8744296; doi:10.1186/s12916-021-02191-2)

**Additional File 2: Figure S1. CV Hospitalization outcome among subgroups**

Multivariate HRs (95% CIs) for factors associated with CV hospitalization among initiators of SGLT2i with DKD and commercial health insurance (N=2,284).


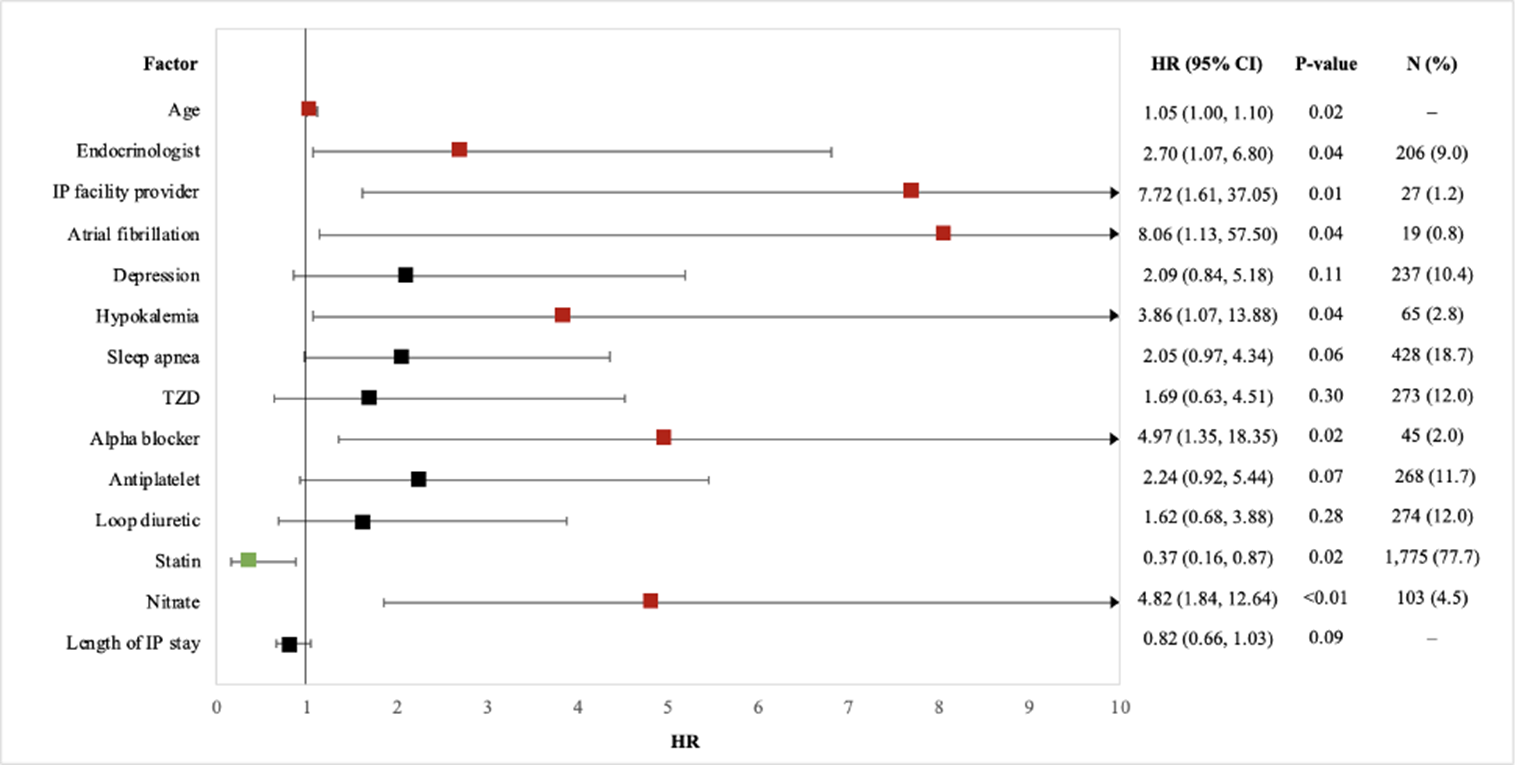


Multivariate HRs (95% CIs) for factors associated with CV hospitalization among initiators of SGLT2i with DKD and Medicare insurance (N=4,105).


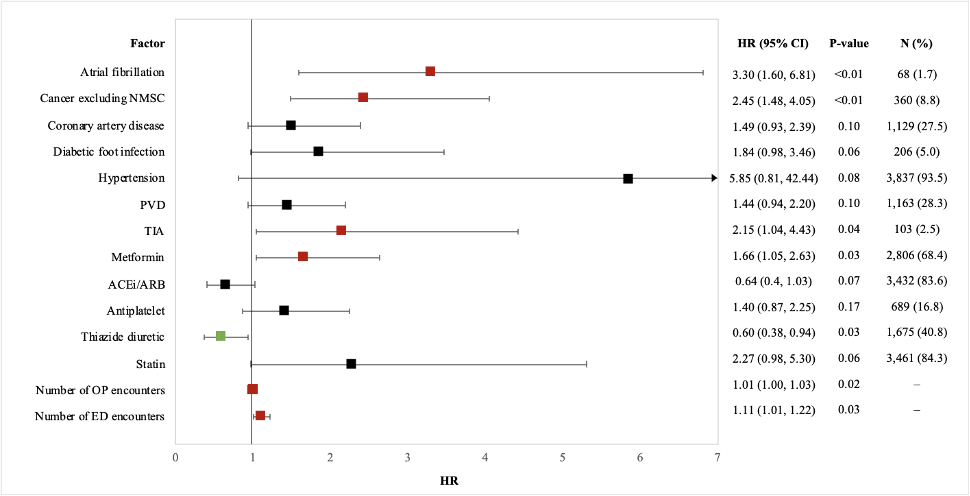


Multivariate HRs (95% CIs) for factors associated with CV hospitalization among initiators of SGLT2i with DKD and low CV risk (N=2,797).


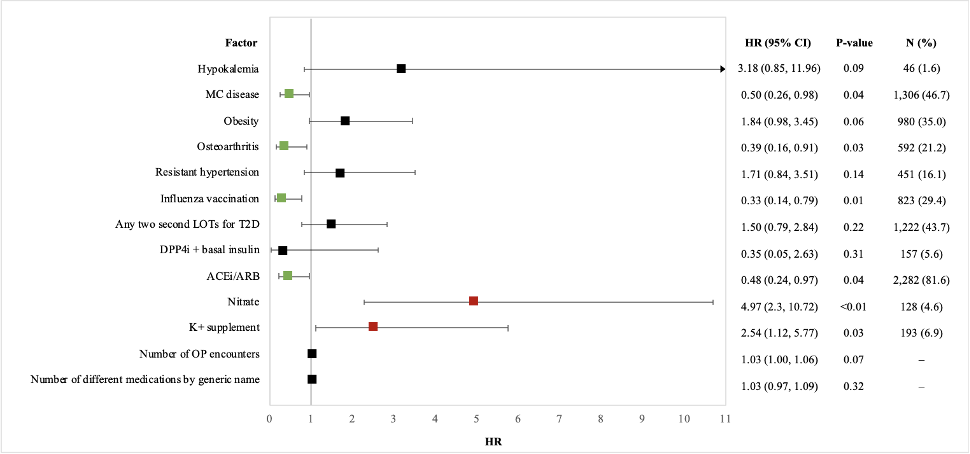


Multivariate HRs (95% CIs) for factors associated with CV hospitalization among initiators of SGLT2i with DKD and moderate CV risk (N=3,237).


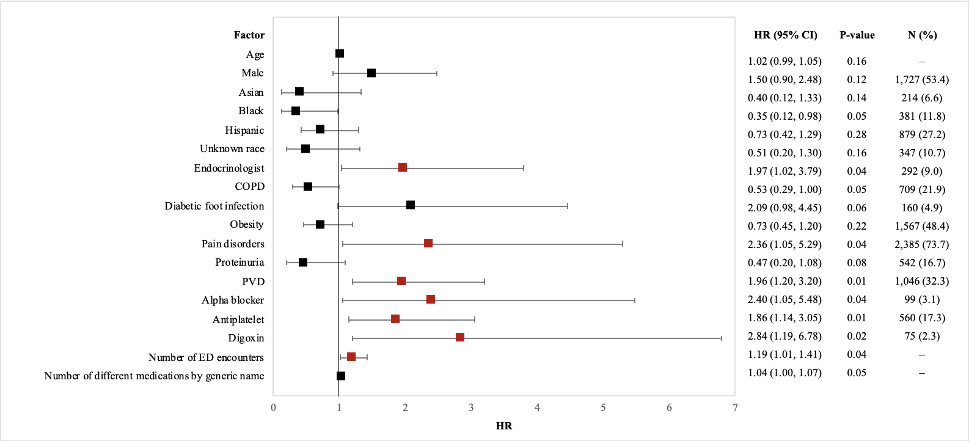


Multivariate HRs (95% CIs) for factors associated with CV hospitalization among initiators of SGLT2i with DKD and high CV risk (N=355).


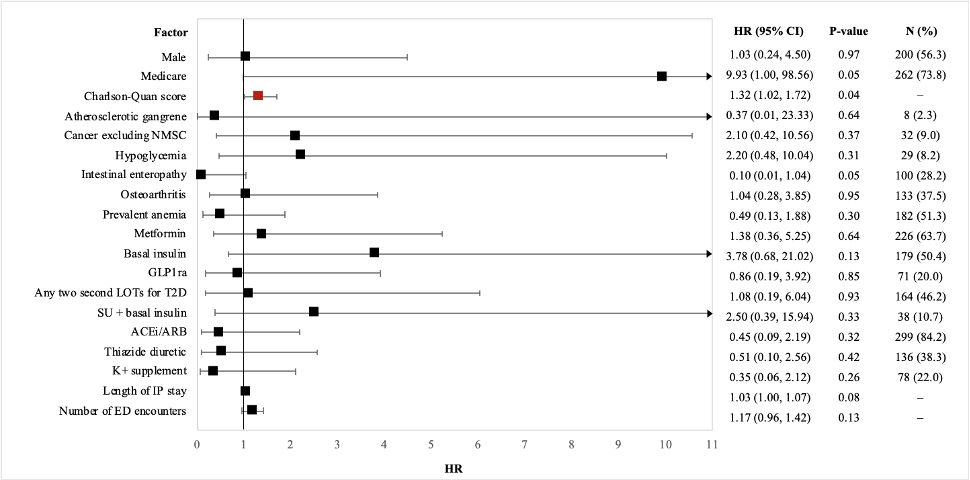


**Additional File 2: Figure S2. Renal Hospitalization among subgroups**

Multivariate HRs (95% CIs) for factors associated with renal hospitalization among initiators of SGLT2i with DKD and commercial health insurance (N=2,284).


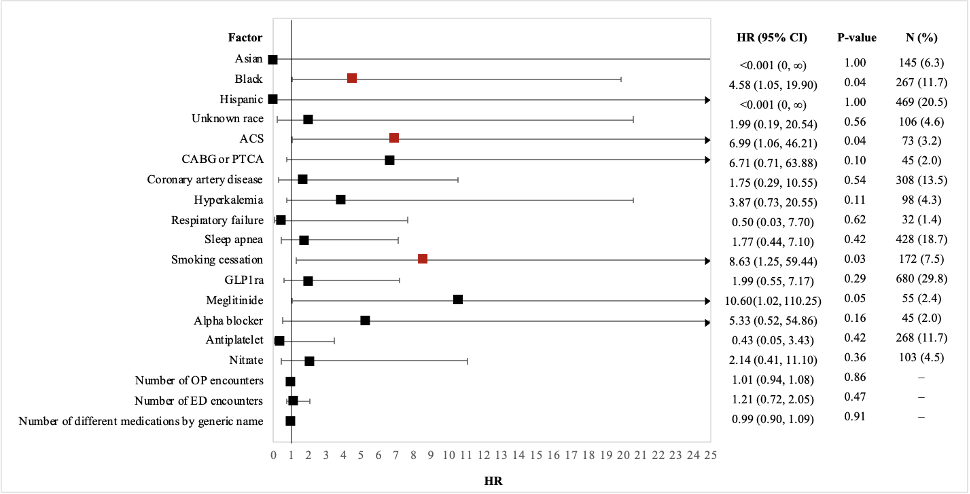


Multivariate HRs (95% CIs) for factors associated with renal hospitalization among initiators of SGLT2i with DKD and Medicare insurance (N=4,105).


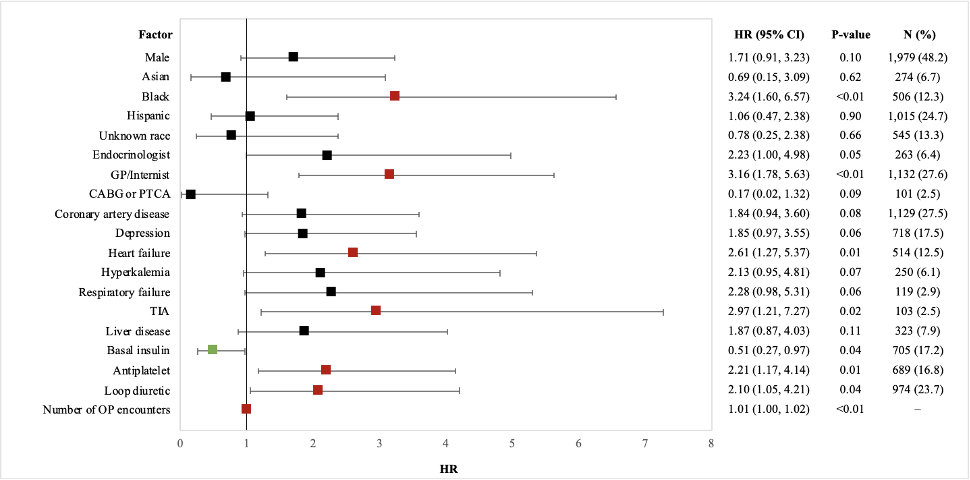


**Additional File 2: Figure S3. AKI Hospitalization among subgroups**

Multivariate HRs (95% CIs) for factors associated with AKI hospitalization among initiators of SGLT2i with DKD and commercial health insurance (N=2,284).


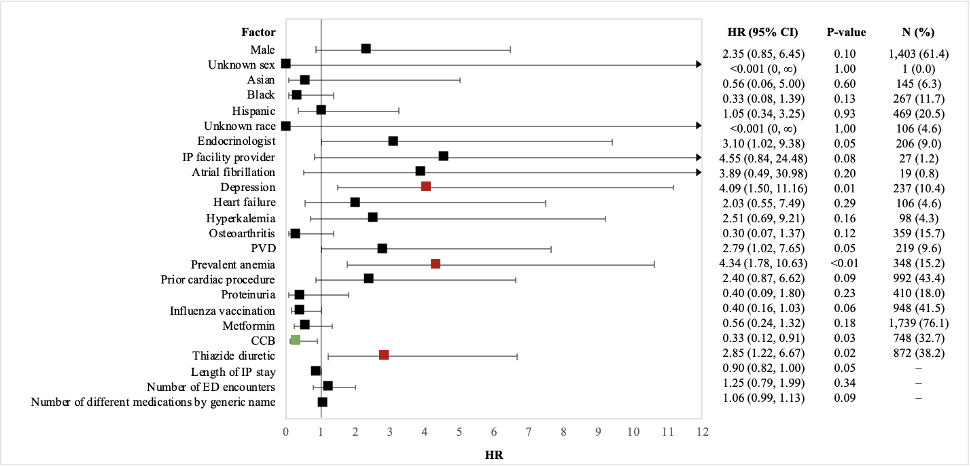


Multivariate HRs (95% CIs) for factors associated with AKI hospitalization among initiators of SGLT2i with DKD and Medicare insurance (N=4,105).


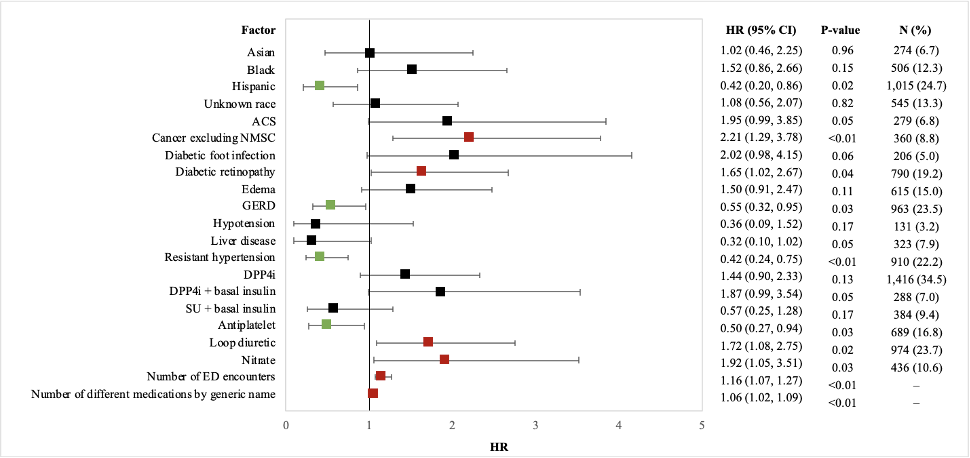


**Additional File 2: Figure S4. Treatment Failure among subgroups**

Multivariate HRs (95% CIs) for factors associated with treatment failure among initiators of SGLT2i with DKD and commercial health insurance (N=2,284).


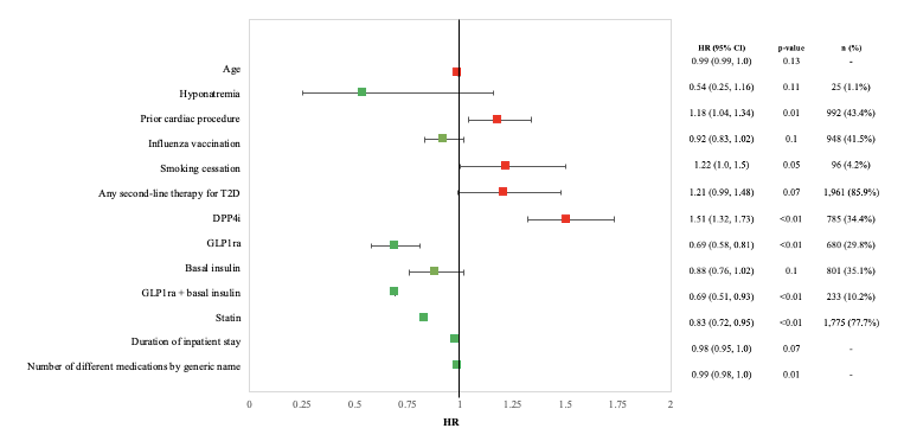


Multivariate HRs (95% CIs) for factors associated with treatment failure among initiators of SGLT2i with DKD and Medicare insurance (N=4,105).


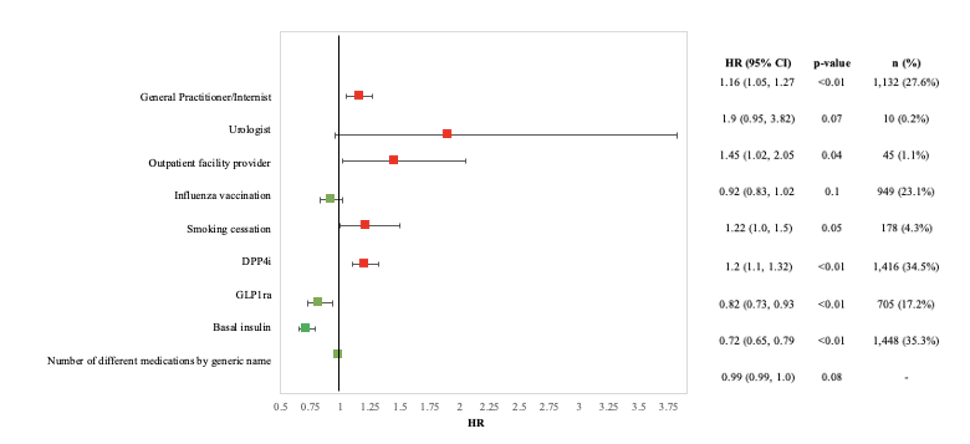

Supplement: Supplementary file 2 — Additional file 2: Figure S1. CV Hospitalization outcome among subgroups. Figure S2. Renal Hospitalization among subgroups. Figure S3. AKI Hospitalization among subgroups. Figure S4. Treatment Failure among subgroups [file 12916_2021_2191_MOESM2_ESM.docx]
